# Local cell interactions and self-amplifying individual cell ingression drive amniote gastrulation

**DOI:** 10.7554/eLife.01817

**Published:** 2014-05-21

**Authors:** Octavian Voiculescu, Lawrence Bodenstein, I-Jun Lau, Claudio D Stern

**Affiliations:** 1 Department of Cell and Developmental Biology, University College London, London, United Kingdom; 2 Division of Pediatric Surgery, Morgan Stanley Children's Hospital of New York-Presbyterian, New York, United States; 3 Department of Surgery, College of Physicians and Surgeons, Columbia University, New York, United States; California Institute of Technology, United States

**Keywords:** epithelial–mesenchymal interactions, EMT, cell movements, primitive streak, modelling, computer simulation, chicken

## Abstract

Gastrulation generates three layers of cells (ectoderm, mesoderm, endoderm) from a single sheet, while large scale cell movements occur across the entire embryo. In amniote (reptiles, birds, mammals) embryos, the deep layers arise by epithelial-to-mesenchymal transition (EMT) at a morphologically stable midline structure, the primitive streak (PS). We know very little about how these events are controlled or how the PS is maintained despite its continuously changing cellular composition. Using the chick, we show that isolated EMT events and ingression of individual cells start well before gastrulation. A Nodal-dependent ‘community effect’ then concentrates and amplifies EMT by positive feedback to form the PS as a zone of massive cell ingression. Computer simulations show that a combination of local cell interactions (EMT and cell intercalation) is sufficient to explain PS formation and the associated complex movements globally across a large epithelial sheet, without the need to invoke long-range signalling.

**DOI:** http://dx.doi.org/10.7554/eLife.01817.001

## eLife digest

A key process during the development of an embryo involves a single layer of cells reorganizing into three ‘germ layers’: the ectoderm, which becomes the skin and nervous system; the mesoderm, which gives rise to the skeleton, muscles and the circulatory and urinogenital systems, and the endoderm, which gives rise to the lining of the gut and associated organs. The process of forming these three layers is known as gastrulation.

To date most experiments on gastrulation in vertebrates have been performed on frog embryos. However, the embryos of amniotes, the group of ‘higher’ vertebrates that comprises reptiles, birds and mammals, differ from those of frogs in a number of ways. Now Voiculescu et al. have used a combination of experimental and computational techniques to shed new light on gastrulation in chick embryos.

Just prior to gastrulation, the cells of the amniote embryo are arranged in a flat disk, one cell thick, called the epiblast. The cells of the epiblast then move to form the mesoderm and endoderm (in a process called epithelial-to-mesenchymal transition). These cell movements also lead to the formation of a structure called the primitive streak that establishes the left-right symmetry of the organism, and also defines the midline of the body.

Now Voiculescu et al. have shown that the epithelial-to-mesenchymal transition starts before the primitive streak appears, and that two main processes drive gastrulation. One involves cells inserting themselves between other cells at the midline of the epiblast, which causes a double whorl-like movement within the plane of the epiblast. At the same time small numbers of cells leave the epiblast, and as these cells accumulate under the epiblast, they initiate a positive feedback effect by which they encourage more cells to leave the epiblast. Voiculescu et al. found that this ‘community effect’ involves signalling by a protein called Nodal. This protein effectively amplifies the epithelial-to-mesenchymal transition and leads to the appearance of the primitive streak at the midline.

Using computational modelling, Voiculescu et al. argue that the movements of gastrulation can be explained entirely based on local interactions between cells, without the need for cells to send signals over long distances to guide cell movements, as had been generally believed.

**DOI:** http://dx.doi.org/10.7554/eLife.01817.002

## Introduction

**Figure 1.**
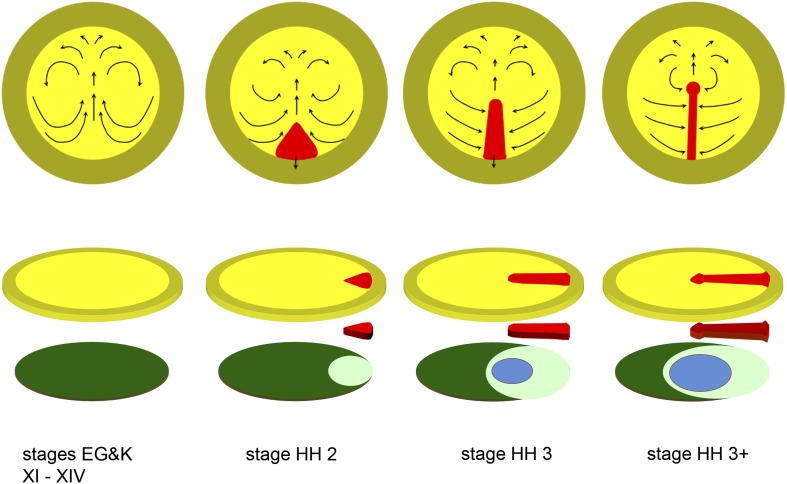
Diagrams depicting the early stages of chick development. The upper row of diagrams shows embryos at stages XI-XIV (pre-primitive streak), 2 (early streak), 3 (mid-streak) and 3^+^ (mid- to late streak), viewed from the dorsal (epiblast) side. The arrows denote the main morphogenetic movements (‘Polonaise’) occurring within the plane of the epiblast. After stage 4 (end of gastrulation), convergence of cells towards and ingression through the anterior part of the streak slows down or ceases (although these movements continue through the middle and posterior parts of the streak), while the epiblast anterior to the streak (prospective neural plate) elongates (Sheng et al., 2003); later, the streak starts to regress, further lengthening the neural plate posteriorly (Spratt, 1947). The lower row of diagrams shows an exploded view of the embryos at each of the above stages, with the top row of diagrams representing the upper layer (epiblast, shades of yellow), the bottom row showing the lower layer (shades of blue/green: hypoblast in dark green, endoblast in light green, definitive or gut endoderm in blue) and the centre row showing the middle (mesodermal) layer (primitive streak, in red). Within the epiblast, the central (yellow) region is the area pellucida and the outer (mustard) region the extraembryonic, area opaca. **DOI:** http://dx.doi.org/10.7554/eLife.01817.003

Before gastrulation, the embryo of reptiles, birds and most mammals is a large flat disc of epithelial cells (epiblast) (Pasteels, 1940). In the chick, the 50,000 or so cells that comprise the embryonic epiblast (area pellucida, 3–5 mm in diameter) move as two bilaterally symmetrical whorls, known as the ‘Polonaise’ pattern (Gräper, 1929; Wetzel, 1929) (Figure 1, stage EGK XI-XIV). The movements continue for 8–10 hr, culminating in the formation of a stable morphological structure in the posterior midline, the primitive streak (PS) (Figure 1, stage HH2). Stage HH2 is very brief, as the PS then quickly narrows and elongates along the midline of the embryo, reaching about 2/3 of the diameter of the area pellucida in a further 8–10 hr (Figure 1, stages HH3 and 3^+^). Once the PS forms, cells in the epiblast lateral to the PS start moving directly into it along trajectories perpendicular to its axis (for reviews see Spratt, 1946; Nicolet, 1971; Bellairs, 1986; Stern, 2004b) (Figure 1, stages HH3-3^+^). The PS acts as a gateway for gastrulation as epiblast cells internalize via epithelial-to-mesenchymal transition (EMT) (Nieto, 2011) and generate mesoderm and endoderm beneath the epiblast layer. At present we do not understand the cellular or molecular mechanisms of any of these events, nor do we know whether they are controlled separately or represent the manifestation of a single underlying process.

Many theories have been proposed to account for the early movements of the epiblast (Table 1 in Chuai and Weijer, 2009). One type of model invokes long-range, diffusible chemotactic attractants or repellents emanating from various parts of the embryo, to which epiblast cells respond as individuals. For example, (Vasiev et al., 2010) suggested that the tip of the PS produces repellents for cells in the rest of the epiblast, while (Sandersius et al., 2011) proposed that the PS acts as a ‘chemotactic dipole’, secreting repellents at the tip and attractors at the base, to which epiblast cells respond. Differential adhesion between cells destined to ingress and the rest of the epiblast is also invoked by some models (Vasiev et al., 2010). All of these models are complicated by the fact that the extracellular matrix, presumed to be the substrate over which the epiblast moves, is secreted by both epiblast and underlying hypoblast and actually moves along with the cells (Harrisson et al., 1985a; Harrisson et al., 1985b; Zamir et al., 2006; Zamir et al., 2008). Some models do not envision the extracellular matrix as a substrate for cell movements. One of these (Wei and Mikawa, 2000) focused on streak elongation, proposing that oriented cell division could drive this process. Another class of mechanism involves epithelial intercalation of epiblast cells at right angles to the future midline in the presumptive domain of the PS, which is initially located along the posterior edge of the epiblast: this could drive the elongation of this domain and may also contribute to the Polonaise movements (Voiculescu et al., 2007). However none of these models is sufficient to account for all four major movements of chick gastrulation: the Polonaise of the early epiblast, the elongation of the PS, the movement of epiblast cells towards the streak and their ingression through the streak (Table 1). To date, only a very complex combination of various unrelated mechanisms, involving oriented cell division in the streak, secretion of signals by the streak that repel its tip (‘mechanism M3’ of Vasiev et al. (2010)), induced cell polarization of the epiblast and differential adhesion of the prospective mesendoderm to neighbouring cells (‘mechanism 11’ of Table 1 in Vasiev et al. (2010)), has come close to delivering the full repetoire of key movement patterns.

**Table 1.** Summary of the four main classes of model (with an example of each) proposed to explain aspects of chick gastrulation **DOI:** http://dx.doi.org/10.7554/eLife.01817.004

Model	Mechanism(s)	PS elongation	Early epiblast movements (Polonaise)	Late epiblast movements	Ingression
Wei and Mikawa, 2000	oriented cell division in PS	**x**			
Bodenstein and Stern, 2005	movement and incorporation of lateral cells into PS +/− active movement within PS	**x**			
Voiculescu, et al., 2007	intercalation in PS region	**x**	**x**		
Vasiev, et al. 2010	repulsion by tip of PS	**x**		**x**	
Sandersius, et al., 2011	repulsion and attraction by PS	**x**		**x**	

The last four columns summarise the specific cell movements that are explained (x) or not explained (blank) by each model. None of the existing classes of model is sufficient by itself to account for all the movements observed.

Several other problems are not addressed by any current model. One of these is how the PS is maintained as a morphologically stable structure despite the fact that cells are continuously moving into and out of it. We know very little about the dynamics of EMT for individual cells, how collective EMT arises and how the PS is maintained as a stable structure despite its constantly changing cellular composition. The PS acts like the blastopore (a canal connecting the outer and inner layers of the embryo) of lower vertebrates, but the PS does not have an obvious opening, raising the question of how cells are internalized through an apparently solid structure. It is also unclear how the epiblast preserves its integrity and characteristic columnar epithelial organisation of cells with apical-basal polarity during this process.

Here we address these questions and provide evidence that the epiblast is highly dynamic and that local cell interactions are sufficient to explain global morphogenetic movements across a large epithelial sheet without the need for long-range signalling.

## Results

**Figure 2.**
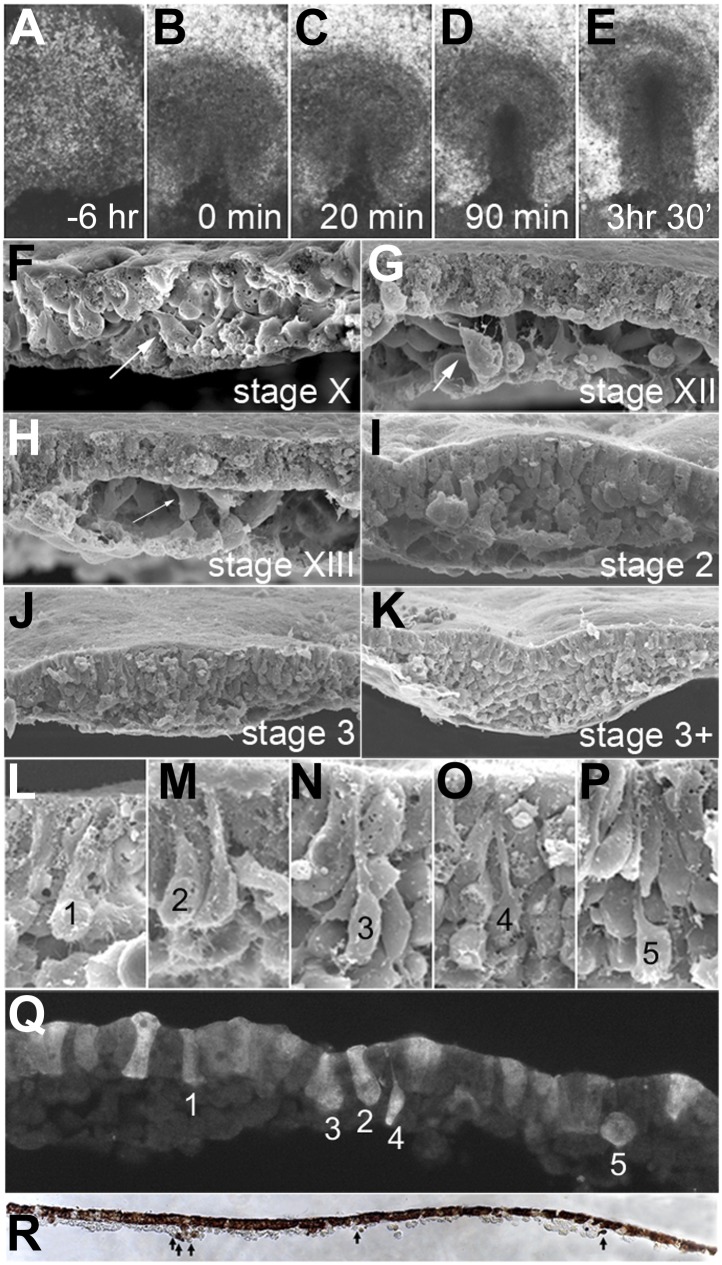
EMT in the formation of the primitive streak (PS). (**A**–**E**) Images from a time-lapse sequence of entire embryos (Video 1), showing the uniform epiblast 6 hr (**A**, stage EG&K XII) and just before primitive streak formation (**B**, stage EG&K XIV), the first appearance of the primitive streak (**C**, stage HH2), accumulation of mesoderm beneath the flat streak (**D**, stage HH3), appearance of a groove in the PS and emigration of mesoderm (**E**, stage HH3+). (**F**–**K)** SEM of fractured embryos before (**F**–**H**) and after (**I**–**K**) streak formation. White arrows indicate possible EMT before PS formation. (**L**–**P**) SEM of fractured PS, showing EMT cells with various degrees of apical constriction and basolateral expansion (classified as ‘ingression stages 1–5’). (**Q**) This embryo was cultured for 1 hr after electroporation of a control, fluorescent morpholino into the entire epiblast at stage XI, then sectioned sagitally and viewed under fluorescence. Labelled cells in the epiblast show similar morphologies to those in SEMs (panels **L**–**P**, ‘ingression stages 1–5’). (**R**) This embryo was cultured for 4 hr after electroporation of a control, fluorescent morpholino into the entire epiblast at stage XI, then fixed (at stage XII), sectioned sagitally and stained with anti-fluorescein antibody (brown). The section shows several cells that have left the epiblast and are now in the underlying space throughout the anterior-posterior extent of the embryo (arrows). **DOI:** http://dx.doi.org/10.7554/eLife.01817.005

Conventional time-lapse video microscopy reveals that the PS appears abruptly, forming a triangular structure within 10–30 min (Figure 2A–E, Video 1). This event defines the transition between stages XIV (Eyal-Giladi and Kochav, 1976) and 2 (Hamburger and Hamilton, 1951) (Figure 1). Scanning Electron Microscopy (SEM) of embryos at successive stages, fractured perpendicular to the forming streak, reveals a growing population of middle layer cells (prospective mesoderm and endoderm) underlying an uninterrupted flat sheet of epiblast (Figure 2F–K). Close to the PS, the epiblast displays cells with various degrees of apical narrowing and baso-lateral expansion (designated 1–5 in Figure 2L–P), indicative of bottle-like cells undergoing EMT. The PS only develops a marked midline groove many hours later, by which time it contains many deep cells and its length has extended to about 2/3 of the diameter of the area pellucida (stage 3^+^, Hamburger and Hamilton, 1951; Figure 1); even then, it does not contain a blastopore-like opening that could act as a portal for gastrulation (Bancroft and Bellairs, 1974; Vakaet, 1984; Figure 2K).

**Video 1.** The primitive streak forms abruptly. Chick embryo development from late blastula to full primitive-streak stages. Time is indicated in hh:mm. The video shows the entire embryo (about 3 mm in diameter) and was made using a 2.5x objective and a conventional upright compound microscope with bright field optics. **DOI:** http://dx.doi.org/10.7554/eLife.01817.006

**Figure 3.**
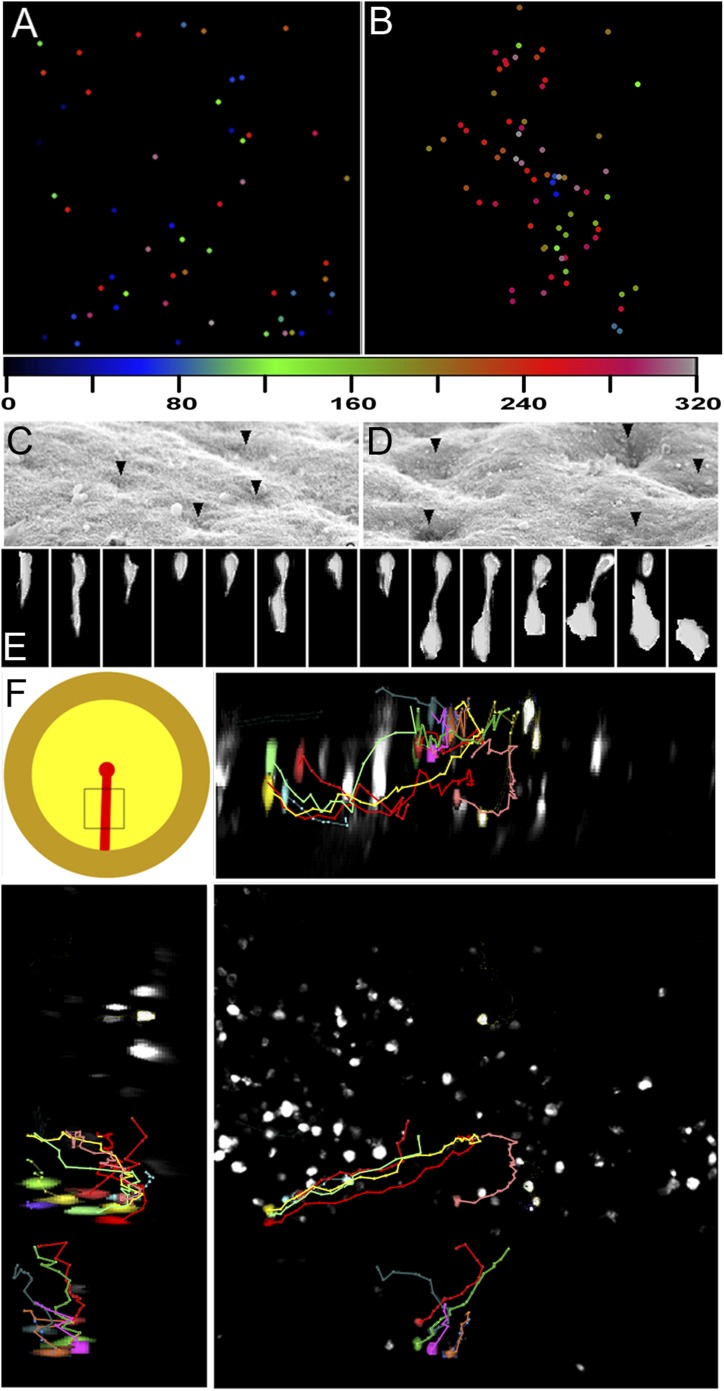
Clustering of seemingly stochastic EMT underpins the formation of PS. (**A** and **B**) Uniform distribution of EMT in the epiblast before PS formation (**A**) and acceleration of EMT as the PS appears (**B**). Locations are plotted from 6 hr time-lapse sequences (see Videos 3 and 4, respectively) and the time of ingression is colour-coded (numbers represent minutes). Each field of view is 600 × 600 μm, in the central posterior epiblast (where the primitive streak arises). (**C** and **D**) Apical surface of the epiblast seen in SEM at PS formation stages. (**E**) An individual epiblast cell followed in time-lapse before (see Video 5) undergoing repeated attempts at full EMT. (**F**) Multi-photon time-lapse sequence of EMT at PS stages. The top left-hand panel shows a diagram of the embryo with the area imaged enclosed in a square. The other panels represent views in the x-z (top right), y-z (bottom-left) and x-y (bottom-right). The positions of selected, colour-coded cells at successive time points (10 min intervals) are connected with lines. **DOI:** http://dx.doi.org/10.7554/eLife.01817.007

To examine when EMT begins, we labelled cells in the epiblast before PS formation (stages X-XIII, Eyal-Giladi and Kochav, 1976) by widespread electroporation of a fluorescein-labelled control Morpholino. Within 1 hr after labelling and at all embryonic stages examined, all regions of the epiblast contain some cells at different stages of EMT: Figure 2Q shows an example, where cells with different morphologies have been classified into five ‘ingression stages’ (1–5) equivalent to those seen by SEM (see above). 4 hr after labelling (Figure 2R), some cells have left the epiblast but can be distinguished from hypoblast cells because the latter are much larger. Multi-photon time-lapse sequences reveal individual ingression events widely distributed in the epiblast, as early as stages X-XII, 6–15 hr before streak formation (Figure 3A; Video 2).

**Video 2.** Isolated EMT in the epiblast before primitive streak formation. Multi-photon time-lapse sequence of the posterior epiblast at pre-primitive streak stages. The embryo was labelled by electroporation of a fluorescein-coupled control morpholino at stage EG&K XI, imaged every 10 min until stage EG&K XII (time indicated in hh:mm). Top view perpendicular to the epiblast (maximum intensity projection, scan depth 100 μm, z-spacing of 3 μm; scanned area 600 μm × 600 μm). Prospective ingressing cells in this sequence are marked by a blue dot; a red dot marks each ingression event. Relates to Figure 2A,B. **DOI:** http://dx.doi.org/10.7554/eLife.01817.008

**Figure 4.**
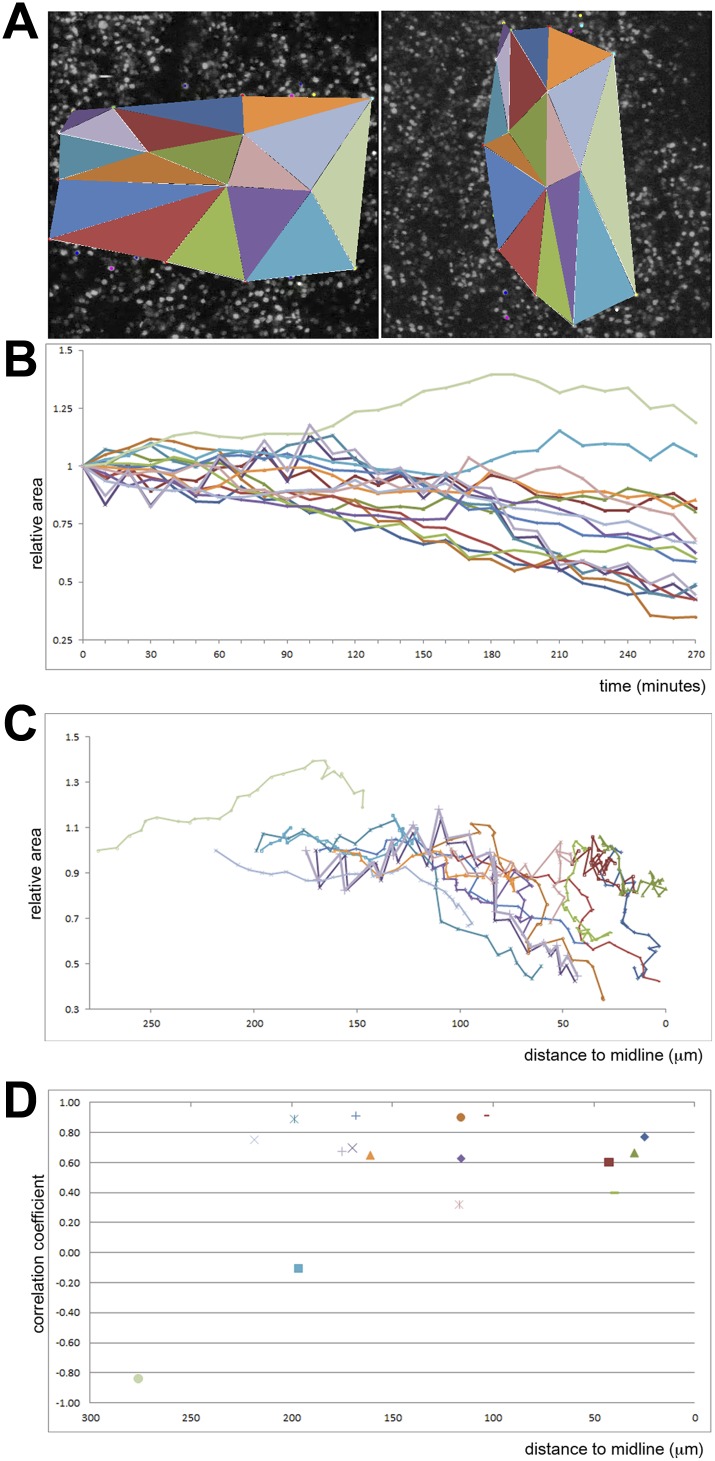
Quantification of ingression from the epiblast with time. Cell ingression accelerates as the PS forms and cells approach its midline. (**A**) the first and last frames of Video 4 (left and right panels, respectively), highlighting the triangles used for measuring. (**B**) relative change in surface area of each triangle over time (min). (**C**) relative change in surface area of each triangle as a function of distance to the midline (in μm). (**D**) correlation coefficient (r^2^) of the size reduction plotted against initial distance to the midline (μm). **DOI:** http://dx.doi.org/10.7554/eLife.01817.009

What underlies the transition from isolated EMT scattered across the epiblast to massive internalization at the PS? Using SEM, we observed that the apical surface of the epiblast displays depressions 3–4 cells wide (Bancroft and Bellairs, 1974) at stages XIII-2 (just before and as the PS appears); these increase in width and depth by stage 3 (Figure 3C,D), suggesting coordinated apical constriction. In multi-photon time-lapse sequences, ingression events can be seen to accelerate as the streak forms (Figure 3B, Figure 4; Videos 3, 4). Individual cells scattered throughout the epiblast undergo repeated cycles of incomplete delamination as they move towards the streak; this continues even at later PS stages (Video 5; Figure 3E). Few cells far from the streak complete their delamination (Video 6). Ingression increases as cells approach the PS so that most of them ingress within 1 hr; however, cells reaching it at the same time do not necessarily ingress synchronously (Video 7; Figure 3B,F) and some ingressions occur away from the PS midline. For example in Figure 3F, a cluster of cells highlighted with different colours at the far left of the lower right hand panel (dorsal view of the epiblast) shows that cells that are close to each other ingress at different times and different positions along their trajectory towards the PS (middle of the panel): one of the red cells (higher in the panel) ingresses furthest from the streak and earliest, whereas the other red cell (the lowest in the group) ingresses only when it reaches the streak. Together, these observations show that ingression of epiblast cells occurs throughout the epiblast at a low rate, but this rate increases markedly in the region of the forming streak.

**Video 3.** EMTs accelerate and cluster at the time of primitive streak formation. Multi-photon time-lapse sequence of the posterior epiblast around the time of primitive streak formation. The embryo was labelled by electroporation of a plasmid driving the expression of H2B-EGFP, imaged every 10 min between stages EG&K XIII and HH 3 (time indicated in hh:mm). The upper panel is a top view of the epiblast (maximum intensity projection, scan depth 100 μm, z-spacing of 3 μm; scanned area 600 μm × 600 μm). The lower panel is a side view (YZ projection, along the forming primitive streak). Prospective ingressing cells are marked by a blue dot in this sequence and a red dot marks each ingression event. Relates to Figure 2A,B and Video 4. **DOI:** http://dx.doi.org/10.7554/eLife.01817.010

**Video 4.** Changes in surface area in regions close to the site of primitive streak formation. The coloured dots mark some cells which do not ingress and which could be followed throughout the sequence in Video 3. 15 triangles were drawn to connect sets of three cells. The surface area of each of these triangles was measured at each time point (every 10 min), and the relative changes used to assess the net rate of ingression in each region; the results are plotted in Figure 4. **DOI:** http://dx.doi.org/10.7554/eLife.01817.011

**Video 5.** Cells attempt EMT several times before full ingression. Multi-photon time-lapse sequence of an embryo at stage HH 3^+^, whose epiblast was electroporated with a plasmid driving DsRed-Express, imaged every 10 min (time indicated in hh:mm). 3D-reconstruction with the basal side of the epiblast towards the viewer and the axis of the primitive streak running from top (anterior) to bottom (posterior). Cells of the wire-frame grid cells are squares 30 μm × 30 μm. One cell attempting EMT is highlighted in green and shown magnified in the insert to the right. **DOI:** http://dx.doi.org/10.7554/eLife.01817.012

**Video 6.** Isolated EMTs outside the primitive streak. Multi-photon imaging of another embryo at stage HH 3^+^, which had been electroporated with DsRed-Express plasmid around the primitive streak and imaged at 10 min intervals (time indicated in hh:mm). 3D-reconstruction with the basal side of the epiblast towards the viewer (similar to the one in Video 5), with the primitive streak running from upper right (posterior) to lower left (anterior). The arrow in the first frame points to a cell which will ingress outside the primitive streak. **DOI:** http://dx.doi.org/10.7554/eLife.01817.013

**Video 7.** EMTs seem stochastic even at full primitive streak stages. Tracking of cell nuclei in an embryo at stage HH 3^+^, which had been electroporated with H2B-EGFP plasmid and imaged at 10 min intervals. Time indicated in hh:mm; the tracks are colour-coded as indicated in the time bar (lower-right; time indicated in hh:mm). The green balls show the positions of chosen nuclei at each time point. To allow visualization of all tracks, two views are shown from slightly different angles in the left and right main panels. In both, the apical side of the epiblast is towards the viewer and its basal side away; the primitive streak runs along the middle (anterior towards the top, posterior towards the bottom). The black insets (top right corner of each main panel) show an overview of the entire volume scanned. Cells of the grid box are squares, 50 mm × 50 mm. **DOI:** http://dx.doi.org/10.7554/eLife.01817.014

**Figure 5.**
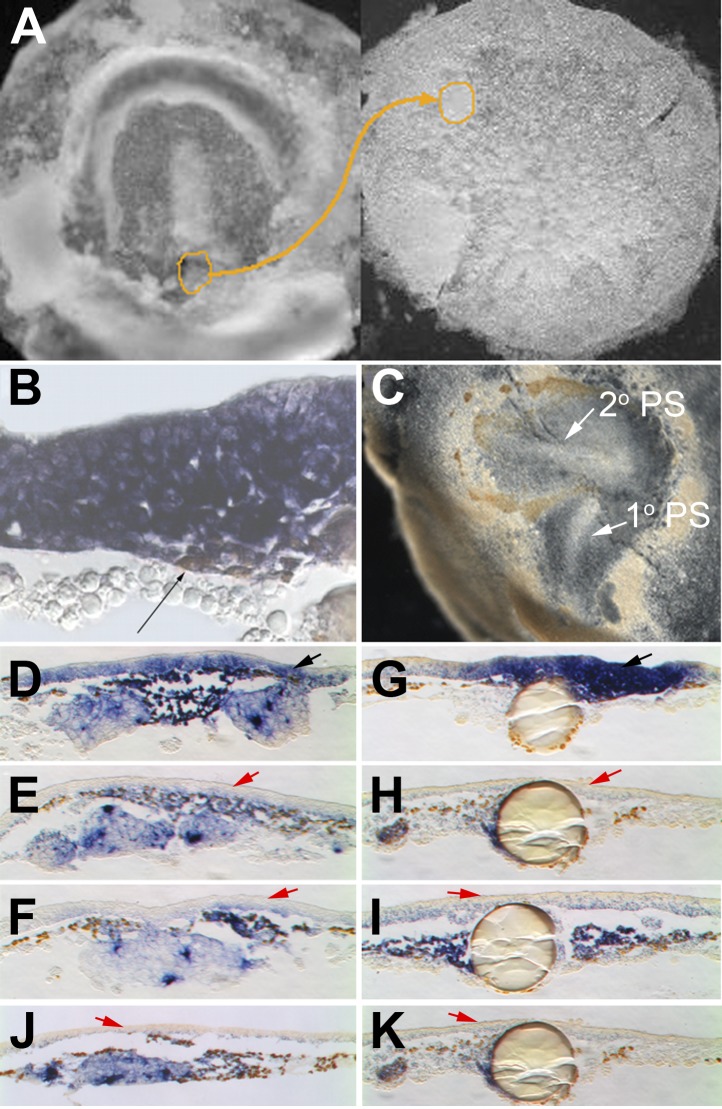
Cells in EMT trigger a chain reaction of EMT in a Nodal-dependent manner. (**A**) EMT cells from the early PS of a quail embryo (left) are grafted under the epiblast of a pre-PS chick embryo (right). (**B**) Grafted cells (brown stain, thin black arrow) upregulate EMT markers (*cSnail2*, purple) and trigger EMT from the epiblast above, after 4 hr. (**C**) Grafted embryo after 15 hr. The grafted quail cells (brown) have migrated away, and the new PS they triggered (‘2^o^ PS’) is composed of host cells. The PS developing along the original orientation is labelled ‘1^o^ PS’. In grafts combined with COS cells secreting Cerberus (**E**) or Cer-S (**F**), or beads soaked in SB431542 (**H**) or SB505124 (**I**), EMT (thickening) from the epiblast and induction of *cSnail2* (purple) in the epiblast (red arrows) are abolished. Control COS cells (**D**) or beads soaked in DMSO (**G**) do not abolish the induction by the grafted mesoderm (black arrows). Mesoderm from a region lateral to the PS cannot induce EMT or *cSnail2* either alone (not shown) or in the presence of GFP-transfected COS cells (**J**) or beads soaked in solvent alone (**K**) (red arrows). **DOI:** http://dx.doi.org/10.7554/eLife.01817.015

The increase in ingression rate in the proximity of the future PS territory suggests that cells that have already ingressed may favour ingression of their neighbours. To test this, we grafted small groups of ingressed cells from the posterior early PS (stage 2–3) of quail embryos, under the epiblast of pre-PS stage chick embryos (stage XII–XIII) (Figure 5A). We chose these cells because they only contribute to lateral mesoderm and not to axial tissue (Hatada and Stern, 1994; Psychoyos and Stern, 1996); this differs from grafts of organizer or Koller's sickle (Izpisua-Belmonte et al., 1993; Bachvarova et al., 1998), both of which contribute to and induce an organizer (Izpisua-Belmonte et al., 1993; Bachvarova et al., 1998; Streit et al., 2000). The grafted cells induce PS markers in the adjacent epiblast within 4 hr (*cBra*, 8/8 embryos, *cSnail2*, 10/10) and massive ingression ensues (Figure 5B). After 14 hr, a second, host-derived streak develops from the graft site (8/11; Figure 5C), whereas the grafted cells (prospective lateral mesoderm [Hatada and Stern, 1994; Psychoyos and Stern, 1996]) migrate away. When a similar graft is made using mesoderm from more lateral cells that have emerged from the PS, no such induction occurs (see below). This is consistent with a previous study (Vakaet, 1973) using full-thickness grafts of posterior mature PS (‘nodus posterior’). Our results implicate the mesoderm as the source of the inductive signals. Early ingressed cells induce mesendodermal identity and increase the probability of other epiblast cells undergoing EMT, suggesting that the PS forms and maintains itself by positive feedback mediated by a community effect (Gurdon, 1988).

What is the molecular basis of this community effect? Candidates include pathways implicated in mesendoderm induction (FGF, TGFβ/Nodal) and/or patterning (canonical Wnt, BMP) (Carnac and Gurdon, 1997; Standley et al., 2001; Stern, 2004a). We co-transplanted recently ingressed cells with COS cells secreting specific inhibitors (Figure 5E–F) or beads soaked in chemical modulators of each pathway (Figure 5H–I). SU5402 (FGF-inhibitor), Crescent, Dkk and alsterpaullone (canonical-Wnt-modulators), chordin and noggin (BMP-inhibitors) did not inhibit induction (n = 9 each except Dkk, n = 7). However, Cerberus (BMP- and Nodal-inhibitor, 9/9; Figure 5E) and Cerberus-Short (Nodal-inhibitor, 8/9; Figure 5F), as well as SB4315412 (10/12; Figure 5H) and SB505124 (11/12; Figure 5I) (inhibitors of TGFβ superfamily receptors ALK4/ALK7) all prevented both the induction of PS markers (*cBra*, *cSnail2*) and ingression of epiblast cells adjacent to the graft. Importantly, they can do this without loss of the markers in the graft cells themselves (Figure 5E–F). Control COS cells (Figure 5D) and beads (Figure 5G) do not prevent induction by the grafted mesoderm. Grafts of mesoderm from outside the PS do not induce the markers either in the presence (Figure 5J,K) or absence (not shown) of beads or COS cells. These results suggest that TGFβ-related factors, and most likely Nodal, are required for the community effect by newly-ingressed mesendoderm. *Nodal* is expressed before streak formation in a posterior domain of the epiblast (Bertocchini and Stern, 2002; Skromne and Stern, 2002), but its activity is initially blocked by Cerberus (Bertocchini and Stern, 2002), an antagonist produced by the hypoblast. This expression domain seems to be identical to the region in which we previously found cells to undergo intercalation parallel to the marginal zone, driven by the Wnt-PCP pathway (Voiculescu et al., 2007). The domain of *Nodal* expression and intercalation adopts the shape of the forming streak.

**Figure 6.**
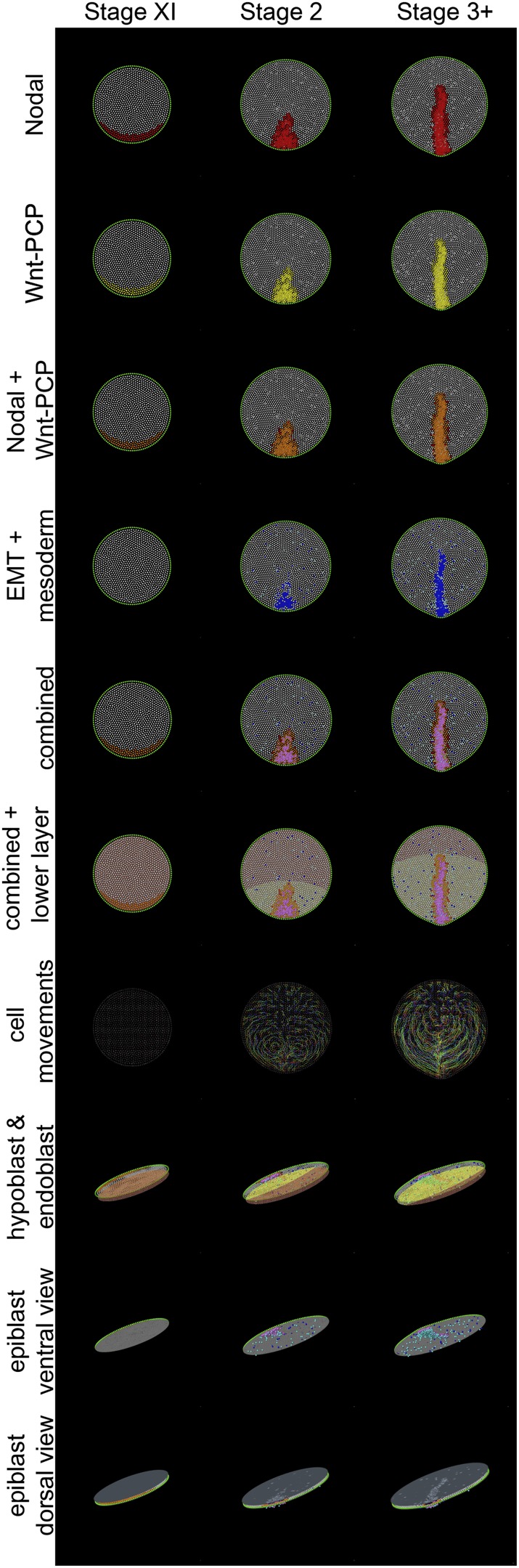
Different views of a simulation of normal development. These diagrams provide an explanatory key for the simulation videos and illustrate the principal signals, cell behaviours and the major tissues involved in gastrulation. Three time points are shown: stage XI, stage 2 and stage 3+. The upper 7 rows are dorsal views onto the epiblast; the lower 3 rows are oblique views. Colours are additive when a cell is positive for more than one displayed state (see e.g., the row labelled ‘combined’, which symbolises the sum of all features in the rows above it for the forming primitive streak). Nodal(+) cells are shown in red (top row), Wnt-PCP(+) cells in yellow (second row). Cells positive for both Nodal and Wnt-PCP appear orange (third row). At Stage XI all cells in the future streak-forming region are Nodal and Wnt-PCP positive. Later, most continue to have both activities but some cells are only positive for Nodal (red). Cells undergoing EMT are shown in blue and ‘mesendodermal’ cells in aquamarine (fourth row). For combinations of Nodal, Wnt-PCP, EMT and mesendoderm, note that Nodal(+)-EMT cells appear purple (red + blue); if also Wnt-PCP(+) then approximately violet (red + yellow + blue) (‘combined’). The hypoblast is shown chocolate-coloured and the endoblast greenish-slate (rows 6 and 8). Hypoblast displacement by the endoblast (at stage XIV; between stages XI and 2 in the Figure) disinhibits Nodal in the overlying epiblast (see text). Sequential cell positions are integrated by remembering all previous time points to form ‘trails’, as shown in row 7. For clarity, trails made from 15% of the cells are shown. The last three rows depict the embryo viewed from an oblique angle. In row 8 (‘hypoblast and endoblast’), the position of the lower layer can be seen (also see above, *lower layer*). Initially this consists only of hypoblast (chocolate). At later stages, endoblast (greenish-slate) partially displaces the hypoblast. The epiblast is also seen from below (‘epiblast ventral view’, row 9), allowing clear visualization of EMT (blue/purple/violet) and emerging and emerging middle layer (aquamarine) cells. The final row, ‘epiblast dorsal view’ (row 10), displays the epiblast from above with a pseudo-surface applied, simulating indentations caused by ingressing cells. These indentations sum as cells approach the posterior midline, generating a midline groove at the PS. The pseudo-surface is created by tessellating points representing the top of each epithelial cell (using the cell body for cells undergoing EMT). **DOI:** http://dx.doi.org/10.7554/eLife.01817.016

Thus, two separable local cell interactions (intercalation and EMT amplified by a community effect) are necessary for PS formation. Are they sufficient to explain PS shape and appearance as well as the complex pattern of tissue movements before and during gastrulation? To address this question we used an agent-based model where these cell behaviours are explicitly added to a simple representation of a bounded epithelial sheet (‘Materials and methods–Description of the model’). The model assigns various states (e.g., Wnt-PCP, Nodal) to cells (Figure 6; Table 2); cells modify their states and execute behaviours based upon their current internal state and interactions with their neighbours (e.g., oriented intercalation, self-amplifying EMT; see Table 3 for a summary of the model rules).

**Figure 6—figure Supplement 1.**
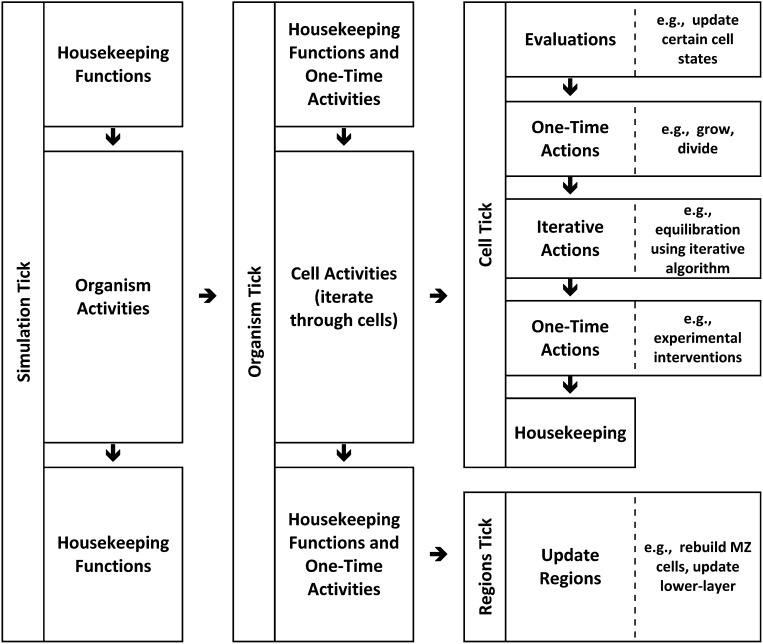
Modelling: hierarchical time implementation. Time is represented as ‘ticks’. Each simulation tick executes activities that include a set of actions for the entire embryo (‘organism tick’). The organism tick in turn executes activities including a cell tick for each cell in the organism. Cell ticks calculate and execute activities for each cell. Note that many of these calculations and activities are themselves iterative. **DOI:** http://dx.doi.org/10.7554/eLife.01817.017

**Figure 6—figure Supplement 2.**
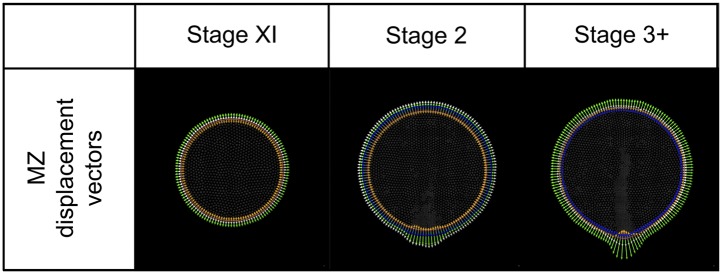
Modelling: MZ displacement vectors. For each MZ cell a displacement vector (white arrowhead) is calculated as the vector sum of ‘curvature’ (orange), ‘density’ (green) and ‘area correction’ (blue) vectors. A mark (red dot) identifies the common origin of each. Vectors are shown magnified 50x for illustration. **DOI:** http://dx.doi.org/10.7554/eLife.01817.018

**Figure 6—figure Supplement 3.**
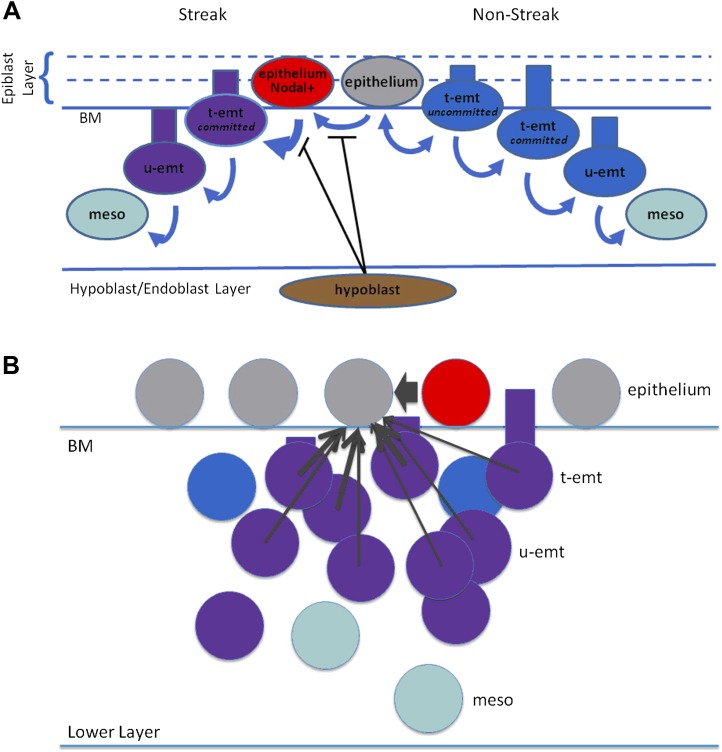
Modelling: schematic representation of EMT and Nodal expression. (**A**) Shown is a schematic representation of EMT in the model. *Right side:* Nodal(−) epithelial cells (grey) may convert to emt cells (blue) which at first are tethered to the epithelium (t-emt) but then become untethered (u-emt) as they descend into the middle layer. They complete the transition as mesenchymal (meso) cells (aquamarine). While still tethered and with cell body above the basement membrane (BM), some will revert and rejoin the epithelium (double-headed arrow). *Left side:* Nodal(−) epithelial cells convert to Nodal(+) (red) in the region of the PS. The rate of EMT increases with increasing Nodal activity from the cell and its neighbours; Nodal-active emt cells (red + blue = purple) lose the ability to rejoin the epithelium (thicker, single-headed arrow). Conversion to Nodal-positivity and the enhanced rate of EMT is inhibited by the hypoblast and disinhibited when the endoblast displaces the hypoblast. (**B**) Shown are cell interactions leading to Nodal expression. Nodal(−) epithelial cells (grey) are converted to Nodal(+) cells by near-neighbour Nodal(+) epithelial cells (red) and local neighbour Nodal(+) emt cells (purple = blue[emt] + red[Nodal]). For local neighbours the effect falls off with distance but is particularly enhanced for near-neighbour epithelial cells (arrow widths). A similar scheme (not shown) applies to Wnt-PCP conversion and to EMT recruitment. Numbers of cells, distances and proportions not to scale. BM: basement membrane, t-emt: tethered emt cell, u-emt: untethered emt cell, meso: mesenchymal cell. **DOI:** http://dx.doi.org/10.7554/eLife.01817.019

**Figure 6—figure Supplement 4.**
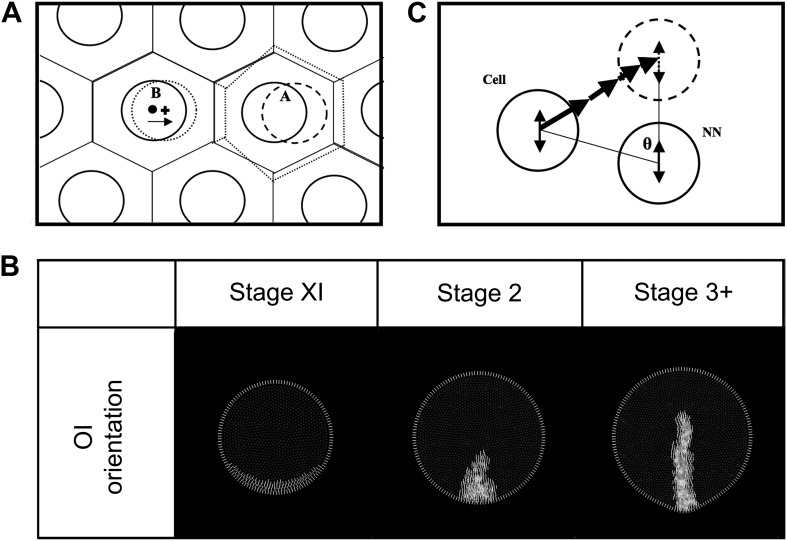
Modelling: cell movements within the plane of the epiblast. (**A**) Schematic diagram of the equilibration algorithm. Cells (solid circles) are distributed in a hexagonal array. Voronoi regions (VR) are transiently generated around these cells (solid lined hexagons). Cell centroids and VR centroids correspond (dot) and the tissue is at equilibrium. When cell ‘A’ shifts to a new position (dashed circle), new VR's are generated (dotted hexagons), making the centroid of the new VR for neighbouring cell ‘B’ move to a new position (cross). Cell ‘B’ then shifts towards this new position to reestablish equilibrium (arrow). The vector from the original centroid of cell ‘B’ to the centroid of the new VR of cell B is the equilibrium displacement vector (**v**_**equil**_). (**B**) Propagation of oriented intercalation orientation vectors**.** MZ-cells maintain a reference OI-orientation vector state perpendicular to the MZ (determined by the local curvature). Epiblast cells calculate their individual OI-orientation vector states by averaging their current vector with the consensus of their near-neighbours, including MZ-cells (see text). Since the MZ-cell vectors are fixed, epiblast cells abutting the MZ will tend to align their vectors to those of the MZ-cells. Note that although this state is stored as a vector in the model, it has angular but not heads vs tails orientation. (**C**) Diagram of the oriented intercalation algorithm. A cell and its near-neighbour (NN) both possess OI-orientation information (double-headed arrows). A sequential displacement vector is calculated, oriented from the cell to its target and with a magnitude equal to |sinθ|. This is applied iteratively for all cells. **DOI:** http://dx.doi.org/10.7554/eLife.01817.020

**Table 2.** List of model cell states which include anatomic cell types as well as signalling mediators and behavioural descriptors **DOI:** http://dx.doi.org/10.7554/eLife.01817.021

	State	Type	Visualization	Description	Initiation, maintenance and propagation
**Cell Types**	**MZ**	boolean	green	MZ pseudo-cells surround the epiblast and form a boundary	initial ring of cells with shape altered to minimize local curvature and epiblast cell density, then adjusted to control epiblast area
	**epiblast**	boolean	grey	epiblast epithelial cells	initial disc of cells; increased by cell division; decreased by ingression
	**emt**	boolean	magenta	epiblast cells undergoing EMT	cells attempting to ingress from the epiblast in a Nodal-dependent process
	**tethered**	boolean	by shape	flags whether or not EMT cell remains tethered to epiblast epithelium	tethered emt cells may re-incorporate into the epithelium whereas untethered cells are committed to progress to mesenchyme
	**meso**	boolean	blue	cells which have completed transition to mesenchyme	end result of EMT; a terminal, inactive state in these simulations
**Other States**	**Nodal**	boolean	red	cells expressing/ secreting Nodal	initially present in PS forming region; cells may be converted to positive by neighbours
	**Wnt-PCP**	boolean	yellow	Wnt-PCP(+) cells capable of oriented intercalation	initially present in the PS forming region; cells may be converted to positive by neighbours
	**OI-vector**	vector	line segment	orients intercalation of Wnt-PCP(+) cells	calculated by consensus among Wnt-PCP(+) cells

**Figure 7.**
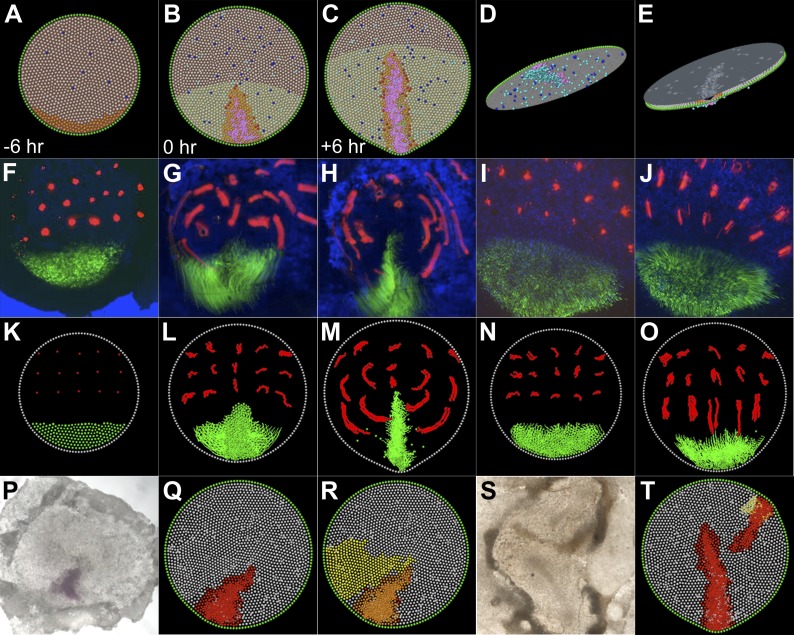
A model based on local cell behaviours explains the global movements in the epiblast and experimental conditions. (**A**–**E**) Epithelial intercalation in a posterior domain (orange) and EMT (blue, isolated events, cooperative in the pink domain) are sufficient to explain the formation of the PS. (**A**–**C**) sequence in time, vertical view; (**D**) ventral view of the epiblast; (**E**) apical view of the epiblast. (**F**–**H**) Sequence from a time-lapse experiment, with cells in the intercalation domain electroporated with control morpholino (green) and other locations in the epiblast labelled with DiI (red). (**F**) initial condition, 6 hr before streak formation; (**G**) movements prior to streak formation; (**H**) movements over 6 hr after PS forms. (**I** and **J**) Movements observed in the same time-frame as in **F**–**H**, when intercalation is blocked by electroporating morpholinos (green) against the Wnt-PCP pathway. (**K**–**O**) The computer model correctly simulates the observed movements both in normal embryos (**K**–**M**) and in intercalation-compromised condition (**N** and **O**). (**P**–**R**) Hypoblast rotation at pre-PS stages leads to bending of the PS. (**P**) Experimental embryo, with the PS marked by *Bra* expression; the model accounts for this result (red in **Q**) by the induction of a new intercalation domain (yellow in **R**) which deforms the original one and the field of cooperative ingression (orange in **R**). (**S** and **T**) EMT cells can trigger a chain reaction of EMT and initiate a new PS in both experimental embryos (**S**) and in the model (**T**). **DOI:** http://dx.doi.org/10.7554/eLife.01817.023

Colour codes can be matched to simulation images in Figures 6 and 7 and Supplementary Videos.

**Table 3.** Description of the rules used in the model **DOI:** http://dx.doi.org/10.7554/eLife.01817.022

**Tissue Structure**
cells in the epiblast are arranged in a flat epithelial layer; cells undergoing EMT descend beneath this layer
the epiblast is surrounded by a ring of marginal zone (MZ) cells that acts as a malleable boundary
**Cell State Activities**
an initial cohort of cells in the posterior epiblast is positive for Nodal and the Wnt-PCP system
Nodal-negative cells may become Nodal-positive if they receive a Nodal signal from neighbours
Wnt-PCP-negative, Nodal-positive cells may be converted to Wnt-PCP-positive if surrounded by Wnt-PCP-positive neighbours
epithelial cells are more likely to undergo EMT if neighbouring cells are undergoing EMT (a community effect mediated in the model by Nodal)
the appearance of the endoblast at Stage XIV displaces the hypoblast (which secretes the Nodal antagonist Cerberus) resulting in Nodal disinhibition in the posterior part of the embryo
**Cell Physical Activities**
epithelial cells undergo a cell cycle and divide in the plane of the epiblast
epithelial cells maintain spatial equilibrium by centring themselves amongst their near-neighbours
epithelial cells may convert to EMT cells which are initially tethered to the epithelium
some EMT cells may become untethered, exit the epithelium and ingress to become mesenchyme
cells not experiencing Nodal activity (either by being far from the primitive streak (PS) where Nodal is expressed, or by having Nodal inhibited by Cerberus from the hypoblast) undergo EMT at a low rate and may revert back to epithelium
Nodal-active epiblast cells undergo EMT at an enhanced rate and do not back-convert
Wnt-PCP-positive cells undergo oriented intercalation with an orientation based on a consensus of the contiguous cohort of intercalating cells, oriented relative to the MZ (intercalation occurs at approximately right angles to the tissue radius)

For details, including mathematical formulations, see ‘Material and methods—Description of the Model’.

In the model, the localized intercalation behaviour, first appearing in the pre-PS epiblast, can recreate movements similar to the early Polonaise seen in real embryos (Figure 7A–E,F–H,K–M; Videos 8, 9); the isolated, uniform EMT occurring at these stages has minimal effect. When cooperativity of EMT is triggered in the intercalation domain (by disinhibition of Nodal activity [Bertocchini and Stern, 2002], because of the displacement of the hypoblast away from the posterior Nodal-expressing zone), massive ingression occurs. In line with experimental observations, this causes the movement pattern to be altered, with cells now entering the PS along direct lateral-to-medial trajectories. The simulations faithfully recreate the large-scale Polonaise movements as well as PS formation and its role as a gateway for gastrulation via cell ingression. Importantly, the global Polonaise movements follow passively from active events localized to the posterior PS-forming region and then the PS itself.

**Figure 7—figure Supplement 1.**
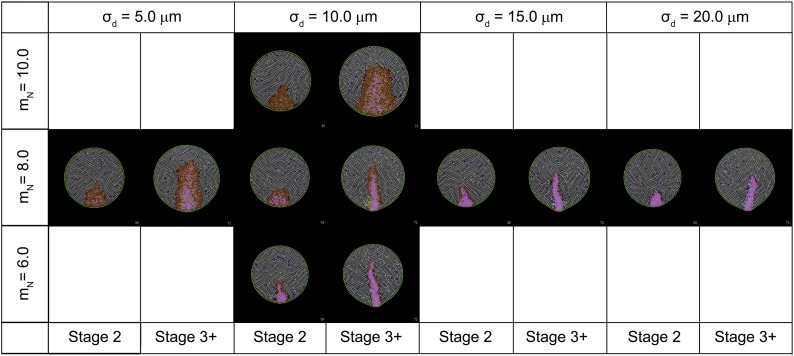
Effect of key parameters on the behaviour of the computer simulation model. The figure shows the composite effects of changing the value of m_N_ and σ_d_ on PS morphology. **DOI:** http://dx.doi.org/10.7554/eLife.01817.024

**Figure 7—figure Supplement 2.**
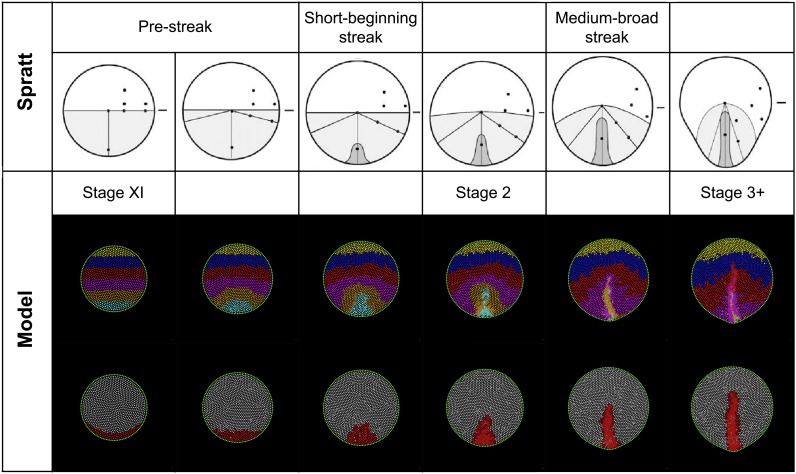
Model predictions compared with the results of Spratt (1946). *Spratt:* global epiblast movements as described by Spratt from carbon-particle marking experiments (Spratt, 1946) (adapted from Spratt 1946). The diagrams combine a movement schematic and a representation of the PS. *Model:* a series of stages in a normal simulation showing the fates of horizontal bands of marked cells (upper row) and formation of the PS (lower row). The simulated pattern is also consistent with more recent analysis of epiblast cell movements and the Polonaise (Foley et al., 2000; Wei and Mikawa, 2000; Voiculescu et al., 2007). **DOI:** http://dx.doi.org/10.7554/eLife.01817.025

**Video 8.** Movements of the epiblast cells before and during gastrulation. Cells in a posterior crescent of the epiblast were electroporated with control morpholino (green), and various locations in the rest of the epiblast labelled with DiI (red) at stage EG&K XII and the embryo filmed in a conventional fluorescence microscope in time-lapse. Time indicated as hh:mm before (negative values) and after primitive streak formation. **DOI:** http://dx.doi.org/10.7554/eLife.01817.026

**Video 9.** Simulation of normal chick gastrulation. Different views of videos showing simulations of normal embryo development (the videos are synchronised with each other). Left column: all cells in the embryonic epiblast are shown in white, confined by the marginal zone (green). In the upper panel, the lower layers are displayed in the background, the hypoblast in pale brown and the endoblast in pale green; in the lower panel, only the epiblast cells are shown. The epiblast cells performing oriented intercalation in the posterior crescent are shown in orange and the early ingressing cells in blue. Cells ingressing by a community effect are displayed in pink. See also Figures 6, 7, Tables 2 and 3, and ‘Materials and methods—Description of the Model for details and colour codes. Middle column: cell movements in the epiblast. In the upper panel, horizontal bands of cells are coloured differently, to allow comparisons with the results in Gräper 1929; in the lower panel, cells in the posterior domain were coloured green and groups of cells in other epiblast locations in red, allowing comparisons with the experimental observations presented here (Video 8). Lower right panel: global movements in the epiblast. A uniform grid of individual objects were tracked over time, and their trajectories are time-coded in rainbow colours. Top right: pattern of ingression in the epiblast. The apical aspect of the epiblast is shown at the top, and its basal side in the lower side of the panel. Cells engaged in EMT are shown in blue, and the locations of completed EMTs in turquoise. Time indicated as hh:mm before (negative values) and after primitive streak formation, as for the experimental observations in Video 8. See also Figure 7F–H,K–J. **DOI:** http://dx.doi.org/10.7554/eLife.01817.027

We tested the effect of changing several parameters. As expected, increasing the strength of intercalation causes the streak to converge and elongate more rapidly, and vice versa. We also examined the kinetics of ingression by changing the mean of the logistic curve defining the probability of ingression (m_N_ in Equation 5.1, ‘Materials and methods–Description of the model’) which effectively alters the community effect threshold and by changing the spatial range of effector cells (σ_d_ in Equation 5.2; d_max_ was co-modified to remain 4 × σ_d_). Figure 7—figure supplement 1 shows images from simulations in which m_N_ is varied from 6.0 to 10.0 (bracketing 8.0 used in the study) and images from simulations where σ_d_ is varied from 5.0 to 20.0 μm (bracketing the 10.0 μm used in this study). Parameter changes that increase the strength of the ingression community effect (smaller m_N_ or larger σ_d_) tend to produce more diminutive streaks since incorporation of new cells into the streak (Equations 6.1, 6.2, 7.1, 7.2) is less able to compensate for increased loss by ingression.

In the model, events in the PS (intercalation and ingression) coupled with the cells in the non-PS epiblast spatially equilibrating amongst themselves results in the global epiblast movement pattern. As the PS converges, cells just lateral and anterior shift posteriorly and medially to ‘fill-in’ the area being evacuated. This shifting is propagated to successively more distant cells. The anterior and lateral epiblast thus sweeps posteriorly and medially. At the same time, convergence and extension in the PS generates movement along the midline; this is mainly directed anteriorly, as the marginal zone limits posterior extension. Together these processes yield both the circumferential (lateral epiblast) and anterior (posterior midline epiblast) components of the Polonaise movement pattern as seen in normal embryos (Spratt, 1946; Figure 7—figure supplement 2).

To distinguish the roles of intercalation and ingression (EMT) in the global Polonaise cell movements, we blocked medio-lateral intercalation by electroporation of a mixture of morpholinos against components of the Wnt-PCP pathway (Voiculescu et al., 2007) and followed the movement of cells elsewhere in the epiblast by using the carbocyanine dye DiI to label a lattice of cells throughout the epiblast (Figure 7I–J). At pre-streak stages, we observe a complete arrest of the Polonaise movements (Gräper, 1929; Wetzel, 1929) across the entire epiblast (Videos 8, 10; in Figure 7 compare G, normal embryo, with I, where Wnt-PCP was blocked in the intercalation domain). Massive ingression is still triggered at the appropriate time, but in a posterior domain close to the margin of the embryo rather than at the midline. In these experiments, cells move posteriorly towards this zone of concentrated ingression along abnormal, straight-line trajectories (in Figure 7 compare H, normal embryo, with J, blocked intercalation). These findings suggest that shaping of the early PS in the normal embryo is mainly driven by intercalation, which results in coalescence and extension in the midline and the Polonaise movement pattern. After its formation, the PS is maintained as a zone of massive ingression that generates a more direct pattern of cell convergence, with cell trajectories perpendicular to the axis of the PS (Video 8; see Figure 1, stage 3+). We used the model to simulate this experiment. With standard parameters and without changing any other conditions, the model faithfully reproduces the altered movement pattern associated with the abrogation of intercalation using Wnt-PCP-Morpholinos in posterior cells (Voiculescu et al., 2007; Video 11; Figure 7N–O).

**Video 10.** The Polonaise movements are driven by intercalation in the posterior domain. Cells in the posterior crescent of the epiblast electroporated with a combination of morpholinos blocking the Wnt-PCP pathway (green), and various locations in the rest of the epiblast labelled with DiI (red) at stage EG&K XII. Embryo filmed by conventional epifluorescence in time-lapse. Time indicated as hh:mm before (negative values) and after primitive streak formation. **DOI:** http://dx.doi.org/10.7554/eLife.01817.028

**Video 11.** Simulation of experimental abrogation of intercalation in the posterior epiblast. In this simulation, oriented intercalation was abolished in 50% of the cells in the domain where this normally occurs (to simulate the Morpholino experiment, Video 10 and Figure 7). Cell states, domains and trajectories are represented in the same way as for the normal embryos, lower row in Video 10. The middle panel allows direct comparison with the experimental blockage of Wnt-PCP pathway in the posterior crescent (Video 10) with electroporated cells depicted in green and dots at random locations labelled in red (to simulate the DiI labelled cells). See also Figure 7I,J,N,O. **DOI:** http://dx.doi.org/10.7554/eLife.01817.029

All above experiments and simulations suggest that just two cell behaviours, cell intercalation and EMT, the latter subsequently amplified locally by a community effect, are sufficient to account for the movements of gastrulation. To test whether they can also account for other reported experimental manipulations, we simulated the effects of hypoblast rotation. In real embryos, rotation of the hypoblast by 90° bends the PS (Waddington, 1932) because of altered cell movements (Foley et al., 2000). Signals from the hypoblast can induce a new domain of PCP activity which was proposed to account for these events (Voiculescu et al., 2007). We used the model to test whether this is a sufficient explanation for bending of the PS; simulations suggest that it is (Video 12; Figure 7P–R). Finally, we tested whether the model can also account for our present finding that an ectopic streak can be induced by a graft of ingressed cells. Again, the model can simulate this result without changing any parameters (see above, Video 13; Figure 7S–T).

**Video 12.** Simulation of hypoblast rotation experiment. Based on experimental findings (Voiculescu et al., 2007), hypoblast rotation by 90° induces a supplementary domain of Wnt-PCP gene expression and oriented intercalation (shown here in yellow) was added to the simulation, to mimic the rotation of hypoblast. As in experimental cases (Waddington, 1932; Foley et al., 2000; Voiculescu et al., 2007), hypoblast rotation leads to bending of the primitive streak. The colour coding follows the scheme employed in Video 9, lower left panel. See also Figure 7P–R. **DOI:** http://dx.doi.org/10.7554/eLife.01817.030

**Video 13.** Simulation of EMT induced by a mesoderm implant. A group of ingressing cells (pink, as in the colour scheme in Video 9, lower left panel) was ‘grafted’ at pre-primitive streak stages to a lateral region of a simulated normal embryo. As in the experiments presented in Figure 5 (see also Figure 7S,T), this results in the induction of an ectopic primitive streak from host cells. **DOI:** http://dx.doi.org/10.7554/eLife.01817.031

In conclusion, our experimental observations and computer model suggest that just two cell behaviours, cell intercalation localized to a posterior domain of the area pellucida epiblast (future streak forming region) together with EMT events, amplified by a community effect mediated by Nodal, are sufficient to explain all four major movements of chick gastrulation: the Polonaise of the early epiblast, the elongation of the PS, the movement of epiblast cells towards the streak and their ingression through the streak.

## Discussion

Several theories have been put forward to account for the origin of the PS and for the associated cell movements. In the chick, some argue that all precursors of the streak arise from and multiply in a very small region of the posterior epiblast (Wei and Mikawa, 2000), whereas others propose that the precursors of the early mesendoderm are present in all regions of the early epiblast, based on the observation that HNK1^+^/Acetylcholinesterase^+^ cells are scattered randomly throughout the pre-PS epiblast (Drews, 1975; Canning and Stern, 1988), contribute to mesendoderm and are required for PS formation (Stern and Canning, 1990). Here we show that ingression of cells starts well before gastrulation, by individual cells sparsely scattered across the epiblast, in a pattern similar to the HNK1^+^ cells. Our results strongly suggest that the early ingressing cells correspond to, or are a subset of the HNK1^+^ population, which also explains why early ingressed mesoderm cells can rescue PS formation in embryos from which HNK1^+^ cells have been ablated (Stern and Canning, 1990). There are interesting parallels with the sea urchin, where gastrulation is initiated by maternally specified ‘pioneer’ cells, the Primary Mesenchyme cells (PMCs) (Sherwood and McClay, 1999; McClay et al., 2000; Sweet et al., 2002), also characterised by their expression of Acetylcholinesterase (Drews, 1975), which carries the HNK1 epitope (Bon et al., 1987; Canning and Stern, 1988). At present, it is not known when and how HNK1+ cells are specified in the chick epiblast, but they share two key characteristics with their sea urchin counterparts: they are required for proper gastrulation (mesendoderm formation) and have inductive abilities. We propose that as in the sea urchin, amniote mesendoderm formation is initiated before the PS forms by ‘pioneer’ cells that ingress as individuals at relatively low frequency throughout the epiblast.

Our results indicate that, in the presence of Nodal activity, these early ingressing cells can trigger a chain reaction of EMT, induce the expression of mesendodermal markers and PS formation. In zebrafish, when the Nodal pathway is compromised, only about 60 cells still ingress instead of the ∼2500 that normally do (Keller et al., 2008), consistent with a community-effect mediated by Nodal being conserved in vertebrates. In sea urchin, however, the inductive effect of PMCs on gastrulation is mediated by Notch signalling (Sherwood and McClay, 1999; McClay et al., 2000; Sweet et al., 2002).

The amniote hypoblast (a transient layer of cells) plays a crucial role in coordinating the timing of PS formation with other cell movements. Medio-lateral intercalation in the epiblast prior to the start of gastrulation acts to displace and re-shape the Nodal domain and the prospective mesendodermal territory to the midline. Later in development, cell intercalation seems to continue to play a role in axial elongation by driving convergence/extension movements in the midline mesoderm and the overlying neuroectoderm (prospective floor plate), after the initial appearance of notochord cells. These later movements in the mesoderm and prospective floor plate are also found in anamniotes, as has been demonstrated in Xenopus and zebrafish (Yeo et al., 2001; Ezin et al., 2006), whereas the early (pre-gastrulation) movements are unique to amniotes. Our results provide a mechanistic explanation for how the displacement of the chick hypoblast (Bertocchini and Stern, 2002) (expressing *Cerberus*) by the endoblast, or of the mouse anterior visceral endoderm (AVE (Perea-Gomez et al., 2002), expressing *Cerberus* and *Lefty1*) leads to extensive EMT and PS formation. We propose that apart from a role in nutrition of the embryo, the hypoblast/AVE acquired the function of delaying PS formation while repositioning the streak precursor cells to the midline (Stern and Downs, 2012). This occurs because the *Nodal*-expressing domain also expresses components of the Wnt-PCP pathway and undergoes intercalation, independently of ingression (Voiculescu et al., 2007). Distinct molecular pathways mediate the dual role of the hypoblast: FGF, which controls the Wnt-PCP pathway and positions the PS (Voiculescu et al., 2007), and Nodal antagonism (perhaps together with Wnt antagonism), which regulates the timing of PS formation (Bertocchini and Stern, 2002).

We suggest the following model of amniote gastrulation. (I) In the stages leading up to gastrulation, cells in a crescent-shaped posterior region of the epiblast express *Nodal* and the Wnt-PCP system. The Wnt-PCP system drives oriented intercalation of epithelial cells, parallel to the marginal zone (perpendicular to the future body axis). Nodal is a potent enhancer of EMT activity (ingression) and sensitizes cells to activity in neighbouring cells (community effect). However, underlying the epiblast is the hypoblast, a suppressor of Nodal activity (through Cerberus). Thus cells outside the posterior region (Nodal-negative) and cells within the region (Nodal-positive but hypoblast suppressed) only attempt EMT at a low rate, and most of these attempts are unsuccessful: they do not result in cell ingression. The Wnt-PCP system causes this posterior cohort of Nodal-positive cells to converge to, and extend along, the midline. Movement of these cells towards the midline draws in neighbouring cells, the displacements are propagated outwards and, through the geometry of the roughly circular epiblast confined within the marginal zone, the Polonaise movement pattern ensues. (II) Endoblast derived from the posterior germ wall (the deep, yolky cells of the area opaca, Stern, 1990) displaces the hypoblast away from the posterior part of the embryo, unleashing Nodal activity in the Nodal-positive population, now localized at the posterior midline. Under the influence of Nodal, ingression accelerates and becomes self-reinforcing, generating the PS. The now massive loss of cells through ingression within the midline PS pulls in lateral neighbours and the displacements are propagated laterally, resulting in a transverse movement pattern. As lateral cells enter the PS, they become Nodal and Wnt-PCP positive and fuel the process. The PS continues to elongate through incorporation of lateral cells and oriented intercalation.

Together, our results suggest that amniote gastrulation is a population event. The PS is not a fixed gateway for cell internalization but rather a dynamic, self-reinforcing concentration of individually ingressing cells. These results provide a mechanism for the self-maintenance of stable morphological structures as their cell composition changes (Joubin and Stern, 1999). They also demonstrate that large-scale movements and morphogenesis of entire epithelial sheets can be driven by local cell interactions, without the need for signalling over long distances.

## Materials and methods

Embryos were cultured by a modification of the New culture technique (Stern and Ireland, 1981). Standard methods were used for electroporation with fluorescein-tagged morpholinos (1 mM) or with pCMV-H2B-EGFP (1 mg/ml, kind gift from K Hadjantonakis) (Voiculescu et al., 2007; Voiculescu et al., 2008) and in situ hybridisation (Streit and Stern, 2001). For multi-photon imaging, fertile chick embryos (Henry Stewart & Co. or Winter Egg Farm, UK) were incubated at 38°C for 2 hr or 14–16 hr, to reach stages EG&K XI (Eyal-Giladi and Kochav, 1976) or early stage HH3^+^ (Hamburger and Hamilton, 1951) respectively, electroporated, reincubated for 4 hr in the case of PS stage embryos, then placed in imaging chambers (Voiculescu and Stern, 2012). Imaging was performed with either a Leica SP2 or MP2 microscope, fitted with a Tsunami XI infrared laser tuned at 895 nm, using a x40 (N.A. = 0.8) lens. Image processing was done using ImageJ, Volocity and Imaris software. For labelling small groups of cells in the embryo, stock CM-DiI (Molecular Probes, 1 mg/ml in ethanol) was diluted 1:20 in 0.25 M sucrose and applied with a finely-drawn microcapillary attached to a mouth tube.

Mesoderm grafts were done essentially as described (Stern and Canning, 1990; Izpisua-Belmonte et al., 1993; Streit et al., 2000). Using insect pins, the endoderm of donor quail embryos at early stage HH3 (Hamburger and Hamilton, 1951) was removed, then the mesoderm underlying the posterior PS was dissected out. The graft was transferred onto the ventral surface of pre-streak stage chick embryos, and inserted into a small pocket made under the hypoblast, either alone or in combination with clumps of COS cells or AG1X2 beads. Aggregates of transfected COS cells (Skromne and Stern, 2001), cut with insect pins to match the size of the grafted mesoderm, or AG1X2 beads soaked in chemical inhibitors (Streit et al., 2000) (25 µM SU5402, 50 µM SB43142, 5 µM SB505124), were placed adjacent to the graft, the saline withdrawn from the glass culture ring, and the embryos immediately incubated at 38°C in culture as described above.

### Description of the model

Nudge++^TM^ (a product of Olana Technologies, Inc. – info@olanatech.com) is an agent-based modelling system designed to study multi-cellular morphogenesis. The current model builds on previously described versions (Bodenstein and Stern, 2005; Fisher and Bodenstein, 2006). Simulated biological cells are the model agents. A two- or three-dimensional simulated tissue is constructed as a cohort of these model cells. Cells execute individual cellular programs leading to actions. The cellular programs reference internal states and external cues, the latter of which may include the states and actions of neighbouring cells. The pooled behaviour of the entire cohort of cells leads to tissue morphogenesis (Bodenstein, 1986).

#### Simulations overview

Computer simulations were run under Redhat Linux on a Dell M4600 multi-core workstation. For the current study, a spherical cell model was used although a cylindrical extension (tether) is added for cells undergoing epithelial-to-mesenchymal transition (EMT) but still attached to the epiblast. Cell–cell interactions are defined by local neighbour relations and by near-neighbour (abutting) relations. Cells in the epithelial layer calculate near-neighbours by transient polygonal constructions (an approximate Voronoi tessellation). Cells independently cycle and divide. They maintain a variety of internal states which may be altered by cell–cell interactions (Figure 6; Tables 2, 3). These states refer to known molecular mediators (e.g., Nodal, Wnt-PCP), or cell types and/or behaviours (e.g., EMT, intercalation orientation). Mediator states generally influence cells on a stochastic basis with probability distributions that are affected by cell interactions.

#### Time and space considerations

The model uses a hierarchical time structure to reconcile simulated events with real events (Figure 6—figure supplement 1). ‘Ticks’ represent the smallest model time unit corresponding to biological real time. During each tick the simulation performs certain global functions; each cell is addressed individually and its actions are based partly on the combined effects of the various influences described above. Many of these actions involve iterative processes. This hierarchical structure generates a checkpoint system whereby various actions that are not necessarily correlated within the model can be coordinated and the model tissue can be time-matched to the biological tissue. This also allows model parameters to be specified in simple real-world terms (e.g., the length of G2 is 30 ± 3 min) without the need to specify details of how the program manages the underlying mechanics. In the current simulations a tick is designed to mimic 10 min of biological time. In similar fashion, the model divides space into ‘clicks’ which correspond to specified real-world dimensions. Unlike ticks, clicks are divisible and space is not quantized beyond the floating-point limits of the computer platform. In the current simulations, a click is defined as 1.0 μm.

#### Embryonic stages

Simulations are designed to model the chick epiblast from about Stage XI (Eyal-Giladi and Kochav, 1976) to about Stage 3+ (Hamburger and Hamilton, 1951) and correspond to about 12 hr of embryonic time. Stage 2 is reached approximately halfway through the simulation.

#### Embryonic layers

The model simulates three embryonic layers (Stern, 2004a) with different levels of detail. The ‘upper layer’ consists of the epiblast proper and marginal zone (MZ). Upper layer cells interact with each other in terms of behaviour (e.g., maintain separation, divide, grow, move) as well as information (e.g., integrating the signals received by a cell and responding appropriately). The ‘middle layer’ is made up of cells that ingress from the epiblast and convert to mesenchyme. Middle layer cells are represented as discrete objects and interact with the overlying epiblast on an informational basis; however, their post-ingression behaviours and movements are not modelled here. The ‘lower layer’ is a schematized representation of the hypoblast/endoblast layer and does not contain model cells. Polygons representing the expanding endoblast and retreating hypoblast are animated over the course of the simulation relative to events occurring in the other layers.

#### Initial conditions

Simulations begin at Stage XI when the chick epiblast is about 3–6 mm across and contains on the order of 25,000–50,000 cells (Stern, 2004a). The initial model epiblast contains about 1000 cells confined to a plane (Figure 6, Stage XI column). A circular array of boundary cells surrounds the epiblast in the same plane and represents the MZ. A cohort of about 90–100 cells in the posterior epiblast is initialized to represent epiblast adjacent to Koller's sickle, or SAE (Bodenstein and Stern, 2005). This area expresses both *Nodal* and the planar cell polarity (PCP) mediators *Prickle-1*, *Flamingo-1* and *Vangl2* and undergoes medio-lateral intercalation (Voiculescu et al., 2007).

Initially the lower layer contains only the hypoblast, represented as a circular domain of similar dimensions as the epiblast; the middle layer is empty. Over time the endoblast partially displaces the hypoblast in the lower layer and the middle layer becomes colonised by cells ingressing from the upper layer (Figure 6).

#### Cell structure, division and growth

Cells are modelled as spheres with a radius of 5 clicks (5.0 μm). Cells ‘swell’ during metaphase and divide symmetrically, with each daughter cell being half the parental volume before growing to full size. In the model, cell division occurs throughout the epiblast at a homogeneous rate corresponding to a mean cell cycle time of 6 hr (Derrick, 1937; Stern, 1979; Sanders et al., 1993; Voiculescu et al., 2007; Chuai and Weijer, 2009). The orientation of cell division is random within the plane of the epiblast.

#### Marginal zone

The MZ consists of a ring of ‘pseudo-cells’ (green in Figure 6) surrounding the active epiblast and forming a boundary. Epiblast cells interact with MZ cells using the standard cell–cell mechanics (see below). MZ cells themselves are provided as a growing boundary: they do not divide but their number is modified as needed to surround a growing epiblast, maintaining their intercellular spacing.

MZ shape is determined by application of a displacement vector to individual MZ pseudo-cells (Figure 6—figure supplement 2). This displacement vector (**v**_**MZ**_) is the sum of a curvature vector (**v**_**curv**_), an epiblast density vector (**v**_**dens**_) and an area correction vector (**v**_**area**_) (Equation 1.1); these vectors are calculated and applied once at the beginning of each tick. The curvature vector (**v**_**curv**_, Equation 1.2) has direction and magnitude based on the local curvature and acts to flatten the MZ ring at each point. The density vector (**v**_**dens**_, Equation 1.3) is directed outwards from the epiblast with a direction based on the local curvature and a magnitude based on the local density of epiblast cells. In essence, a high local cell density abutting the MZ will push the MZ outwards in that location. The area correction vector (**v**_**area**_, Equation 1.4) is calculated to manage overall cell density of the epiblast despite local changes in the shape of the MZ. The curvature vector is in line with the density vector and either subtracts (if the MZ is locally convex) or adds (if locally concave). (Equations 1.1–1.4). Overall cell density rises slowly during the simulations by use of a logarithmic function, which becomes less prominent at later stages when tissue growth by cell division is mitigated by significant ingression (Equation 1.5).

(1.1) $$v_{MZ}=v_{curv}+v_{dens}+v_{area}$$

(1.2) $$v_{curv}=k_{curv}\kappa {\hat{v}}_{local}$$

(1.3) $$v_{dens}=k_{dens}\left(\overset{N\left(d<d_{\text{max}}\right)}{\underset{i=1}{\sum }}e^{-d_{i}^{2}/\left(2\sigma_{d}^{2}\right)}\right){\hat{v}}_{out}$$

(1.4) $$v_{area}=f\left(A_{t}^{epi}\right){\hat{v}}_{out}$$

(1.5) $$A_{t}^{epi}=\text{max}\left(A_{t-1}^{epi},\left(A_{t}^{cells}/A_{0}^{cells}\right)A_{0}^{epi}\left(1-k_{area}\log_{10}\left(1+9t/k_{t}\right)\right)\right)$$

where, ν_local_ = unit normal (directed towards curvature center), ν_out_ = unit normal (directed outwards from epiblast), κ = local curvature, k_curv_, k_dens_ = 70.0, 0.10, f(area) = iterative function that adjusts epiblast area, N(d < d_max_) = number of epiblast cells at distance d < d_max_, d_i_ = distance of *i*th cell (centre-to-centre), d_max_ = 55.0 μm, σ_d_ = 15.0 μm, $A_{0}^{epi}$ $A_{t}^{epi}$= epiblast area at times 0 and t, respectively, $A_{0}^{cells}$ $A_{t}^{cells}$ = summed cross-sectional area of epiblast cells at times 0 and t, respectively, k_area_, k_t_ = 0.15, 4.0 hr, t = time from simulation initiation (hr).

#### Cell states

Nudge++^TM^ allows creation of an arbitrary number of cell ‘states’ which may be binary flags, quantitative variables or more complex types such as vectors or larger data structures. Several cell states have been defined for these simulations (Table 2, Figure 6). Some states represent descriptors of specific cell behaviours (i.e., orientation of intercalation) or expression of a particular gene (e.g., *Nodal*). Other states refer to an anatomically defined cohort of cells (e.g., MZ) or cell type (e.g., mesenchyme). In the current simulations, daughters inherit their parental states when cells divide (Sarge and Park-Sarge, 2005).

##### Mediator states

A binary ‘**Nodal**’ state marks cells that express/secrete Nodal. Among other actions the model associates active Nodal with an increased propensity for cells to undergo EMT (see below). Nodal activity is inhibited by the hypoblast, which secretes Cerberus (Bertocchini and Stern, 2002), a protein that binds to and antagonises Nodal in the intercellular space (Piccolo et al., 1999). The model integrates the effects of Nodal activity at the receiving, or target, cell. Cells that express/secrete Nodal are deemed *Nodal-positive*. Cells capable of responding to Nodal (e.g., not inhibited by the hypoblast) are *Nodal-receptive*. Nodal-receptive cells actually encountering Nodal and performing Nodal-dependent activities are *Nodal-active*. The corresponding ‘off’ states are *Nodal-negative*, *Nodal-refractory* and *Nodal-inactive*. Note that Nodal-receptive and Nodal-active cells need not be Nodal-positive. Nodal-refractory cells may retain some receptivity, albeit markedly reduced, and Nodal-inactive cells may show some response, albeit much muted. A cell inhibited by the hypoblast (and hence Nodal-refractory) will have a reduced or absent response to Nodal signalling regardless of whether it is Nodal-positive itself.

A binary ‘**Wnt-PCP**’ state marks cells that express components of the Wnt- planar cell polarity system such as *FMI1* (*flamingo-1*), *PRICKLE1* (*prickle-1*) and *VANGL2* (*vanGogh-like-2*). Here, the single Wnt-PCP state represents the combined active pathway. Cells that are Wnt-PCP-positive undergo oriented intercalation (OI) in association with their Wnt-PCP-positive near-neighbours (Voiculescu et al., 2007).

A vector ‘**OI-orientation**’ state is used by Wnt-PCP-positive cells in association with Wnt-PCP-positive near-neighbours to establish the direction of local intercalation (see below).

##### Cell-type states

A binary ‘**MZ**’ state defines pseudo-cells that encircle the epiblast and form a boundary.

A binary ‘**epithelium**’ state defines cells that form the epiblast epithelial layer. Epithelial cell interactions can distinguish near-neighbours (abutting in the epiblast plane) from other local neighbours.

A binary ‘**emt**’ (EMT) state defines cells that initiate ingression and attempt to leave the plane of the epiblast to descend into the deeper layers (Figure 6—figure supplement 3A). These cells may remain tethered to neighbouring epiblast cells (**t-emt**) or break free, become untethered (**u-emt**) and then convert to mesenchyme. EMT cells maintain the Nodal and Wnt-PCP states of the epithelial cell from which they derive but lose positivity if they become mesenchyme.

A binary ‘**tethered**’ state flags whether or not an EMT cell maintains an attachment to the epithelial plane. While EMT cells remain tethered they display an up-and-down motion, often changing direction, until they either re-incorporate into the epithelium or break free and enter the middle layer. The probability of changing direction is modelled using a logistic function with different parameters for descending and ascending cells (Equation 2.1). As cells descend further from the epithelial plane (more negative z-value) their bias to continue downwards increases, and, if moving upwards, they become more likely to change direction.

(2.1) $$p_{\Delta dir}=p_{\text{min}}+\left(p_{\text{max}}-p_{\text{min}}\right)/\left(1+e^{-\left(z-m_{z}\right)/s_{z}}\right)$$

where, p_Δdir_ = probability/hr of a directional change, z = depth of cell from epithelial plane (z = 0.0 μm), m_z_ = −5.0 μm, s_z_ = 1.0 μm, p_min_, p_max_ = 0.8, 0.0 per hr (for cells moving upwards), p_min_, p_max_ = 0.0, 0.4 per hr (for cells moving downwards).

Tethered EMT cells may re-incorporate into the epithelium. To reverse EMT, some of the cell body must be above the BM and Nodal activity must be below a threshold (defined below). For cells eligible to re-incorporate, the probability of re-incorporation follows a logistic function that decreases with increasing distance from the epiblast plane (Equation 3.1).

(3.1) $$p_{emt->epi}=p_{\text{min}}+\left(p_{\text{max}}-p_{\text{min}}\right)/\left(1+e^{-\left(z-m_{z}\right)/s_{z}}\right)$$

where, p_emt->epi_ = probability/hr of reversion of t-emt to epithelial cell, z = depth of cell centre from epithelial plane (z = 0.0 μm), p_min_, p_max_ = 0.0, 0.0 per hr (for z < −10 or untethered or Nodal activity ≥ threshold) [= prohibited], p_min_, p_max_ = 0.30, 0.90 per hr (for z >=−10 and tethered and Nodal activity < threshold), m_z_ = −5.0 μm, s_z_ = 1.5 μm.

EMT cells initially remain tethered to the epithelium (t-emt). They remain so as long as at least some of the cell body is above the BM (z >−10.0 um). Once completely below the BM, the probability of untethering increases with increasing depth according to a logistic function (Equation 4.1).

(4.1) $$p_{untether}=p_{\text{min}}+\left(p_{\text{max}}-p_{\text{min}}\right)/\left(1+e^{-\left(z-m_{z}\right)/s_{z}}\right)$$

where, p_*untether*_ = probability/hr of untethering of emt cell (t-emt to u-emt), z = depth of cell centre from epithelial plane (z = 0.0 μm), p_min_, p_max_ = 0.0, 0.0 per hr (z >=−10 μm) [= prohibited], p_min_, p_max_ = 0.0, 0.999 per hr (z <−10 μm), m_z_ = −12.5 μm, s_z_ = 1.0 μm.

A binary ‘**meso**’ (mesenchyme, or mesendoderm) state defines a cell type that has fully converted from epithelium (epiblast) to mesenchyme by EMT. In these simulations, meso cells have no interacting states and mesenchyme cell behaviours are not modelled.

#### Cell–cell interactions

Cells perform activities or modify their states based upon an internal calculus and interactions with their neighbours—*effector cells* produce an *effector* that influences the *receiving* (or *target*) *cell*. Generally these interactions bias the probability of some action (e.g., change in state, new behaviour), which then occurs on a stochastic basis (Losick and Desplan, 2008).

##### EMT conversion

In the model, conversion of epithelial cells to EMT cells is enhanced by Nodal. This is formulated using a logistic function with ‘effective neighbour equivalents’ (N_eff_) as the dependent variable (Equation 5.1). N_eff_ in turn includes components representing the cell itself, near-neighbours in the epiblast plane and more distant local neighbours whose effect is attenuated with distance (Equation 5.2). Three classes of Nodal-secreting effector cells are distinguished: (i) the receiving cell itself (if already secreting Nodal); (ii) *near-neighbour* cells (those directly abutting the receiving cell in the epithelial layer), and (iii) *local neighbour* cells (those within some proximity, excluding near-neighbours). For local neighbours the effect falls off with distance. Each of these classes is assigned a coefficient that modulates their relative effect. Inhibitors may impact the effect of each of these classes by modification of the coefficients. Inhibition of Nodal by Cerberus from the hypoblast (Bertocchini and Stern, 2002) is modelled by decreasing the coefficient for the neighbour effects when the hypoblast is present, the decrease in the local neighbour effect being greater than that of the near-neighbour effect. This formulation is designed to be consistent with the action of hypoblast derived Cerberus [Bertocchini and Stern, 2002] which binds to and blocks Nodal in the extracellular space [Piccolo et al., 1999].

(5.1) $$p_{emt}=p_{\text{min}}+\left(p_{\text{max}}-p_{\text{min}}\right)/\left(1+e^{-\left(N_{eff}-m_{N}\right)/s_{N}}\right)$$

(5.2) $$N_{eff}=k_{self}N_{self}+k_{nn}N_{nn}+k_{n}\overset{N_{n}\left(d<d_{\text{max}}\right)}{\underset{i=1}{\sum }}e^{-d_{i}^{2}/\left(2\sigma_{d}^{2}\right)}$$

where, p_emt_ = probability/hr of conversion of epithelial to t-emt cell, N_eff_ = neighbour effect, N_self_ = 0 (Nodal-negative), N_self_ = 1 (Nodal-positive), N_nn_ = number of Nodal-positive near-neighbours, N_n_(d < d_0_) = number of Nodal-positive neighbours of distance d < d_max_, excluding N_nn_, d_i_ = distance of *i*th neighbour (centre-to-centre), p_min_, p_max_ = 0.02, 0.999 per hr, m_N_ = 8.0, s_N_ = 1.0, k_self_ = 1.0 (Nodal-active), 0.6 (Nodal-inactive), k_nn_ = 1.0 (Nodal-active), 0.6 (Nodal-inactive), k_n_ = 1.0 (Nodal-active), 0.0 (Nodal-inactive), d_max_ = 40.0 μm, σ_d_ = 10.0 μm.

In the model, epithelial cells initiate EMT on a stochastic basis, but epithelial cells in proximity to other cells undergoing EMT are more likely to undergo EMT themselves, a community effect (Gurdon, 1988). This community effect on cell ingression is modelled using Nodal as the direct effector. However the model can accommodate one or more other effectors that become active either secondary to Nodal action or by a mechanism involving disinhibition after the hypoblast becomes replaced by endoblast. The base rate of ingression is low for cells in Nodal-negative regions (anterior and lateral epiblast) or Nodal-positive but refractory regions (the PS forming region prior to hypoblast withdrawal). When the hypoblast withdraws, cells in the PS region become Nodal-active and the rate of ingression increases. As the process continues, Nodal-positive ingressing cells accumulate beneath the epithelium and cooperativity accelerates. As the steep region of the logistic curve is reached, massive ingression occurs. The influx and incorporation of lateral epiblast into this zone of massive ingression fuels the process and helps to keep it localized. Nodal-active t-emt cells where exposure to Nodal is at or beyond a threshold are prohibited from rejoining the epithelium, as are cells where the cell body has descended fully beneath the BM. The threshold is chosen as the magnitude of the Nodal effect that a Nodal-positive cell would have on itself in the absence of hypoblast inhibition. Thus, reversion is prohibited for disinhibited Nodal-positive cells, but also for disinhibited Nodal-negative cells if surrounded by numerous Nodal-positive neighbours. This mechanism contributes to the enhanced rate of ingression at the PS.

##### Nodal conversion

Nodal also stimulates Nodal production in neighbouring cells (6.1–6.2, Figure 6—figure supplement 3B). Cerberus from the hypoblast decreases this effect. As with EMT, this is modelled by a decrease in the effect of near-neighbours (k_nn_ 1.0→0.6) and elimination of the local neighbour effect (k_n_ 1.0→0.0).

(6.1) $$p_{Nodal}=p_{\text{min}}+\left(p_{\text{max}}-p_{\text{min}}\right)/\left(1+e^{-\left(N_{eff}-m_{N}\right)/s_{N}}\right)$$

(6.2) $$N_{eff}=k_{nn}N_{nn}+k_{n}\overset{N_{n}\left(d<d_{\text{max}}\right)}{\underset{i=1}{\sum }}e^{-d_{i}^{2}/\left(2\sigma_{d}^{2}\right)}$$

where, p_Nodal_ = probability/hr of conversion of epithelial cell from Nodal-negative to Nodal-positive, N_eff_ = effective neighbour equivalents, N_nn_ = number of Nodal-positive epithelial or t-emt near-neighbours, N_n_(d < d_max_) = number of Nodal-positive local neighbours of distance, d < d_max_, excluding N_nn_, d_i_ = distance of *i*th neighbour (centre-to-centre), p_min_, p_max_ = 0.0, 0.999 per hr, m_N_ = 2.0, s_N_ = 0.25, k_nn_ = 1.0 (without hypoblast inhibition), 0.6 (with inhibition), k_n_ = 1.0 (without hypoblast inhibition), 0.0 (with inhibition), d_max_ = 40.0 μm, σ_d_ = 10.0 μm.

##### Wnt-PCP conversion

A strategy analogous to that for Nodal conversion is used to model the effect of Wnt-PCP positive cells on Wnt-PCP negative neighbours (Equations 7.1.–.Equations 7.2). In the model, epithelial cells must also be Nodal-positive to become Wnt-PCP-positive under normal conditions. Although the threshold is lower for Wnt-PCP conversion than Nodal conversion (m_N_ of 1.0 vs 2.0), Wnt-PCP conversion is limited by this requirement for prior Nodal-positivity.

(7.1) $$p_{Wnt-PCP}=p_{\text{min}}+\left(p_{\text{max}}-p_{\text{min}}\right)/\left(1+e^{-\left(N_{eff}-m_{N}\right)/s_{N}}\right)$$

(7.2) $$N_{eff}=k_{nn}N_{nn}+k_{n}\overset{N_{n}\left(d<d_{\text{max}}\right)}{\underset{i=1}{\sum }}e^{-d_{i}^{2}/\left(2\sigma_{d}^{2}\right)}$$

where, p_*Wnt-PCP*_ = probability/hr of conversion of Nodal-positive epithelial cell from Wnt-PCP-negative to Wnt-PCP-positive, N_eff_ = effective neighbour equivalents, N_nn_ = number of Wnt-PCP-positive epithelial/t-emt near-neighbours, N_n_(d < d_max_) = number of Wnt-PCP-positive local neighbours of distance d < d_max_, excluding N_nn_, d_i_ = distance of *i*th neighbour (centre-to-centre), p_min_, p_max_ = 0.0, 0.999 per hr, m_N_ = 1.0, s_N_ = 0.25, k_nn_ = 1.0 (without hypoblast inhibition), k_nn_ = 0.6 (with inhibition), k_n_ = 1.0 (without hypoblast inhibition), k_n_ = 0.0 (with inhibition), d_max_ = 40.0 μm, σ_d_ = 10.0 μm.

##### Oriented intercalation coordination

Unlike cell–cell interactions involving Nodal and Wnt-PCP, which include contributions from local neighbours, the alignment of oriented intercalation orientation vectors (as described below) uses a juxtacrine strategy where only near-neighbours are considered.

#### Planar cell movements

The model uses two algorithms to define the movement of cells within the plane of the epiblast.

##### Equilibration (or centring)

Non-MZ epiblast cells (i.e., epithelium or t-emt) are displaced or move according to an equilibration vector, which serves to centre cells among near-neighbours. For each cell, a polygon is calculated such that each edge is perpendicular to and passes though a line connecting the centre of the cell with that of its near-neighbour and bisects the separation between the cells. This polygon is roughly analogous to a Voronoi region (VR) and is termed the pseudo-Voronoi region (pseudo-VR). Voronoi regions often are termed ‘cells’. To avoid confusion with biological cells, the term region will be used here. For a true Voronoi region, each edge should maintain equal distance from the physical cell [Okabe et al., 2000]. For biological cells schematized as variable size spheres (circles in two-dimensions) the edges would therefore be curved (Li et al., 2006). The straight-sided polygons used here are simplifications adequate for the proposed cell behaviours and computationally simpler. The centroid of the pseudo-VR is then determined and a displacement vector created from the current cell centroid to the polygon centroid (Figure 6—figure supplement 4A). A single near-neighbour is highlighted in this diagram, but all near-neighbours are involved. Each near-neighbour movement causes its own pseudo-VR to change and the process is propagated across the tissue. Although here cells begin in a regular array, cells in the model are not so constrained. Application of this vector acts to move the cell to a more ‘central’ location among its near-neighbours and when applied to the cell population in iterative fashion tends to make cell density homogeneous throughout the tissue. This is recognizable as a form of Lloyd’s algorithm or Voronoi iteration (Du et al., 1999).

Lloyd's algorithm is asymptotic and customarily run to some arbitrary convergence criterion. In the current simulations, OI continuously alters the positions of a subset of cells in the posterior epiblast. Since OI and equilibration (Voronoi iteration, or VI) occur simultaneously, the latter never approaches equilibrium—there is no fixed endpoint and the non-OI (i.e., anterior and lateral epiblast) cells are continuously ‘playing catch-up’. Hence the algorithm is run for a fixed number of iterations (1000 per tick) rather than to some equilibrium criterion.

##### Oriented intercalation

An ‘OI-orientation’ vector state is maintained for cells programmed to undergo intercalation (i.e., Wnt-PCP-positive). During each tick, the vector for each cell is re-calculated as the normalized sum of the cell's current OI-orientation vector and the normalized sum of the OI-orientation vectors of the cell's near-neighbours. This gives equal weight to the cell's current vector and the consensus orientation of its neighbours. Iterative re-calculation of these vectors is done until a stable arrangement is reached among the Wnt-PCP-positive cells (consensus).

When Wnt-PCP-positive cells divide, each daughter inherits both the positive Wnt-PCP state and the OI-orientation vector of the parent cell, the latter then being refined by interaction with its near-neighbours. By the same mechanism, newly Wnt-PCP-positive cells are seamlessly incorporated into the existing cohort.

MZ cells maintain a reference OI-orientation vector derived from the local curvature. Wnt-PCP cells abutting the MZ cue off of these MZ cells and this acts to orient the contiguous cohort of Wnt-PCP cells (Figure 6—figure supplement 4B). Intercalation uses an OI-movement vector (**v**_**oi**_) for each Wnt-PCP-positive cell, which is re-calculated ‘on the fly’ as a function of the position of the cell, that of its near-neighbours and their various OI-orientation vectors (Figure 6—figure supplement 4C). Both the cell and at least one of its near-neighbours must be Wnt-PCP positive for this interaction to occur.

The magnitude of the **v**_**oi**_ vector is equal to |sinθ|; thus, displacement ceases when (if) the target is reached (θ = 0) and is maximum if the cells are side-by-side (furthest from the ideal position). Only one near-neighbour is shown for simplicity, but the displacement vectors to all near-neighbours are summed to produce the final oriented intercalation displacement vector. Since the algorithm is applied iteratively to all cells, during the each iteration the near-neighbour(s) will also move according to the same algorithm. Thus, rather than the cell moving completely to align with near-neighbours, the cell and its neighbours will both incrementally move towards an equilibrium position. In the illustration both cell and near-neighbour have parallel orientation vectors although this is not necessary (or even common) in the simulations.

Iterative application of this vector-directed movement causes neighbouring cells to realign with each other and produces convergence and extension of the involved population (Wallingford et al., 2002; Keller and Davidson, 2004; Voiculescu et al., 2007).
